# Identification and Characterization of Long Noncoding RNAs in Ovine Skeletal Muscle

**DOI:** 10.3390/ani8070127

**Published:** 2018-07-23

**Authors:** Qing Li, Ruizao Liu, Huijing Zhao, Ran Di, Zengkui Lu, Enmin Liu, Yuqin Wang, Mingxing Chu, Caihong Wei

**Affiliations:** 1 Institute of Animal Sciences, Chinese Academy of Agricultural Sciences, Beijing 100193, China; liqing_0507@163.com (Q.L.); rzliu1988@sina.com (R.L.); zhhj1998927@163.com (H.Z.); dirangirl@163.com (R.D.); 15294157518@163.com (Z.L.); hardliubut@163.com (E.L.); 2 College of Animal Science and Technology, Gansu Agriculture University, Lanzhou 730070, China; 3 College of Animal Science and Technology, Henan University of Science and Technology, Luoyang 471023, China; wangyq6836@163.com

**Keywords:** muscle development, lncRNA, sheep, differential expression

## Abstract

**Simple Summary:**

LncRNAs may play important role in many biological processes. The aims of this research were to identify potential lncRNAs active in skeletal muscle of the Texel and Ujumqin sheep and investigate their functions. Overall, 2002 lncRNA transcripts were found, some of which may be related to muscle development. The findings obtained here should promote understanding of the regulatory functions of lncRNAs in ovine muscle development and potentially also in other mammals.

**Abstract:**

Long noncoding RNAs (lncRNAs) are increasingly being recognized as key regulators in many cellular processes. However, few reports of them in livestock have been published. Here, we describe the identification and characterization of lncRNAs in ovine skeletal muscle. Eight libraries were constructed from the gastrocnemius muscle of fetal (days 85 and 120), newborn and adult Texel and Ujumqin sheep. The 2002 identified transcripts shared some characteristics, such as their number of exons, length and distribution. We also identified some coding genes near these lncRNA transcripts, which are particularly associated with transcriptional regulation- and development-related processes, suggesting that the lncRNAs are associated with muscle development. In addition, in pairwise comparisons between the libraries of the same stage in different breeds, a total of 967 transcripts were differentially expressed but just 15 differentially expressed lncRNAs were common to all stages. Among them, we found that TCONS_00013201 exhibited higher expression in Ujumqin samples, while TCONS_00006187 and TCONS_00083104 were higher in Texel samples. Moreover, TCONS_00044801, TCONS_00008482 and TCONS_00102859 were almost completely absent from Ujumqin samples. Our results suggest that differences in the expression of these lncRNAs may be associated with the muscular differences observed between Texel and Ujumqin sheep breeds.

## 1. Introduction

Long noncoding RNAs (lncRNAs) are transcripts longer than 200 nucleotides (nt) that lack protein-coding potential [1]. However, a small number of lncRNAs have been found to encode small proteins [2,3]. Previously, these transcripts were considered as “junk RNA.” But lncRNAs are increasingly being recognized as key regulators of gene expression at multiple levels. With the development of advanced sequencing technologies, more and more lncRNAs have been discovered in *Homo sapiens* [4,5,6]—*Mus musculus* [7,8], *Bos Taurus* [9,10] and *Sus scrofa* [11,12]. Recently, lncRNAs have also been demonstrated to participate in biological progress such as development [13,14,15], cell differentiation [16], transcriptional regulation [17] and disease [13,18]. Some lncRNAs were also reported to play important roles in muscle development [19,20,21] but there are few reports on the characterization of lncRNAs involved in the skeletal muscle development of *Ovis aries*. Many domestic animals, especially some domestic mammal, are raised mainly for meat production in our animal husbandry. Thus, it is of vital interest to reveal the molecular mechanisms underneath skeletal muscle formation and development. We hope to explain these complex processes from a new perspective.

Texel sheep are typically “double-muscle” sheep, while Ujumqin sheep are characterized by fleshy-fat mutton. Thus, these two ovine breeds are suitable models to study muscle development. The maximum myofiber complement of a muscle is achieved prior to birth in sheep. The number of myofibers was nearly constant after birth. Postnatal skeletal muscle development mainly depends on an increase of myofiber cross-sectional area. According to our previous study, the surges in myofiber hyperplasia occurred at 85 days and 120 days in Texel sheep, whereas a unique proliferative surge appeared at 100 d in Ujumqin sheep [22]. Second and latter half of gestation were important. Existing research suggests that the difference in muscular traits between these two breeds is due to gene mutations [23,24] and the influence of microRNAs [25]. However, information about skeletal muscle development-related lncRNAs in sheep is limited. Building on previous studies, we thus investigated the role of lncRNAs in muscle development between Texel and Ujumqin sheep.

The identification and characterization of lncRNAs in ovine (Texel and Ujumqin) gastrocnemius muscle as reported here were achieved using Illumina HiSeq™2000. This led to a total of 2002 lncRNA transcripts being identified and 967 lncRNA transcripts were found to be significantly differentially expressed at four representative stages. The RNA-seq data and collection of novel lncRNA transcripts should contribute to understanding the function and regulation of lncRNAs in sheep, as well as provide an important resource for investigating skeletal muscle development in mammals.

## 2. Materials and Methods

### 2.1. Sample Collection

Ewes of the Texel and Ujumqin breeds were selected based on age (3–5 years old), body weight (50–55 kg) and body size. The estrus cycle of the ewes was synchronized and artificial insemination was completed. The date of artificial fertilization was used as day zero of gestation. The gastrocnemius muscles were collected from Texel and Ujumqin ewes at the following stages: fetuses at days 85 and 120 of gestation, newborn lambs and one-year-old ewes. The samples were dissected into 0.5 m^3^ cubes and treated with RNAlater overnight at 4 °C, followed by storage in liquid nitrogen for RNA extraction. All experimental procedures involving sheep were approved by the Science Research Department (in charge of animal welfare issue) of the Institute of Animal Sciences, Chinese Academy of Agricultural Sciences (IAS-CAAS) (Beijing, China). Ethical approval on animal survival was given by the animal ethics committee of IAS-CAAS (No. IASCAAS-AE-03, 12 December 2016).

### 2.2. RNA Extraction

Total RNA was separately isolated and purified from frozen gastrocnemius samples using an Animal Tissue RNA Purification Kit (Product #TRK-1002; Qiagen, Hilden, Germany) with DNaseI treatment to remove genomic DNA. After agarose gel electrophoresis, two 28S/18S rRNA bands were clearly seen, with no other bands. The total RNA integrity was assessed using an Agilent 2100 Bioanalyzer (Agilent Technologies, Palo Alto, CA, USA), only the samples with RNA Integrity Number (RIN) scores > 8 were used for sequencing.

### 2.3. Library Construction and Deep Sequencing

Equal quantities of total RNA isolated from three gastrocnemius muscle samples of a breed at the same stage were mixed to form a pool. We defined the eight pools as follows: Texel (T) 1 (day 85); T2 (day 120); T3 (neonatal); T4 (adult); Ujumqin (U) 1 (day 85); U2 (day 120); U3 (neonatal); and U4 (adult). Eight RNA libraries were generated from each Texel and Ujumqin sample sets. These eight purified cDNA libraries were used for cluster generation with Illumina’s Cluster Station and then sequenced by Nextomic Corporation (Wuhan, China). The extracted sequencing reads were then analyzed.

### 2.4. Transcriptome Assembly

Raw reads were cleaned by rejecting reads containing adapters, then removing reads containing over 10% poly(N), low-quality reads (>50% of bases with Phred quality scores ≤ 5) and reads with average Phred quality scores < 15. The Phred scores (Q20) and GC content of the clean data were calculated (Table S1). All clean reads were inspected and approved for quality using FastQC (http://www.bioinformatics.babraham.ac.uk/projects/fastqc/, File S2). The detailed criteria that we used to determine that a sequence was of high quality were as follows: the average quality score per base was above 30 and the proportion of reads that obtained more than 35 quality scores was up to 90%. Subsequently, the clean reads were mapped to the ovine reference genome (Oar_v3.1, downloaded from the NCBI database) using TopHat v2.0.14 (Table S2). The mapped reads were assembled with Cufflinks v2.2.1.

### 2.5. Pipeline for the Identification of Multiple-Exon LncRNA

**Figure 1:**
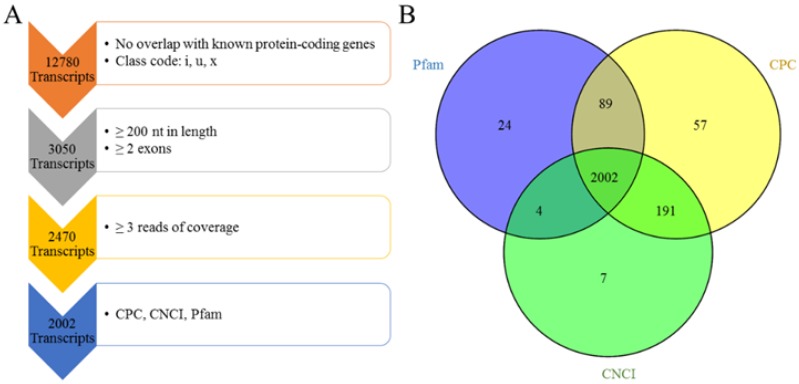
Pipeline for the identification of multiple-exon lncRNA. (**A**) Overview of the stringent filtering pipeline used to identify the resulting 2002 lncRNAs. At each step, the vertical arrow denotes the transcripts that passed the filter and the box denotes the screening conditions. Each step is described in detail in the Methods section. (**B**) The intersection results of CPC, CNCI and Pfam. A total of 2119 lncRNA transcripts were obtained by Pfam, 2339 lncRNA transcripts were obtained by CPC and 2204 lncRNA transcripts were obtained by CNCI. Finally, 2002 lncRNA transcripts were identified from the intersection of the analysis results of CPC, CNCI and Pfam.

The assembled transcripts were filtered following the steps listed in the pipeline depicted in Figure 1. The details of this are as follows: (1) The known ovine genes were removed. Using Cuffcompare, only transcripts annotated as “I,” “u,” and “x” representing novel intronic, intergenic and antisense transcripts, respectively, were retained. (2) Transcripts shorter than 200 bp and transcripts comprising a single exon were removed. (3) The read coverage of every transcript was calculated using Cufflinks v2.1.1. Transcripts with coverage of fewer than three reads were removed. (4) Coding Potential Calculator (CPC) (score < 0), Coding-Non-Coding Index (CNCI) (score < 0) and Pfam scan (E-value < 0.001) were used to assess the coding potential of transcripts. The intersection of the results of each analysis was the set of novel lncRNA transcripts described in our study.

### 2.6. Expression Analysis

The expression level of lncRNA transcripts in each library was estimated using fragments per kilobase of transcript per million mapped reads (FPKM) and differential expression was calculated using Cuffdiff v2.1.1. Q-value < 0.005 and the absolute value of log_2_(fold change) > 1 were set as the thresholds for defining the differential expression of lncRNAs.

### 2.7. Target Gene Prediction and Functional Enrichment Analysis

LncRNAs can act in a cis manner on their neighboring target genes [26,27]. We thus searched for coding genes within 10/100 kb upstream or downstream of all identified lncRNA transcripts and then predicted the functions of these target genes. The names of genes formed a list for WEGO [28] to conduct Gene Ontology (GO) analysis. GO terms with a corrected *p*-value ≤ 0.05 were considered to be significantly enriched. KEGG enrichment analysis of target genes was performed using the KEGG [29] database with a hypergeometric test. KEGG pathways with FDR ≤ 0.05 were considered to be significantly enriched.

### 2.8. Functional Verification of TCONS_00102859 in Myoblasts

#### 2.8.1. Cell Culture and Cell Transfection

Primary myoblasts were isolated from gastrocnemius muscle of 70-day-old ovine fetus. The cells were cultured in DMEM supplemented with 10% fetal bovine serum (FBS) at 37 °C in a humidified 5% CO_2_ incubator and seeded on 12-well plates. Then, the cells were transfected with GV287-TCONS_00102859, which was provided by Shanghai Jikai Corporation. Cells transfected with GV287-N empty vector were set as controls. About 3–4 days after transfection, the expression could be observed. The transfection efficiency was determined by the level of GFP reporter gene expression using a fluorescent inverted microscope. Myoblasts were harvested for RT-PCR analysis 72 h after transfection. RNA extraction and removal of gDNA were performed as described previously. The expression level of GFP reporter gene and qPCR result of TCONS_00102895 were used to decide whether TCONS_00102895 express successfully and steadily.

#### 2.8.2. Reverse Transcription and qPCR Analysis

Reverse transcription was conducted in accordance with the instructions of Prime Script II 1st strand kit. cDNA could be used for downstream analysis. To analyze the expression of TCONS_00102859 in various muscles at different stages, the forelimb skeletal muscle (scapula muscle, triceps) and hindlimb skeletal muscle (longissimus dorsi, gastrocnemius muscle, quadriceps femoris) of 85-day-old fetus and neonate in the Texel and Ujumqin breeds were collected for RT-PCR to determine the expression of TCONS_00102859 in these muscles. Three biological replicates for every stage and every breed were collected. In addition, to investigate the influence of TCONS_00102859 overexpression, qPCR was conducted on cells after transfection to determine the relative expression level of Myogenic D (MyoD), Myogenic G (MyoG), Myocyte enhancer factor-2c (Mef2c) and Myostatin (MSTN), which are related to muscle development. GAPDH was set as a reference gene. The experimental primer pairs for TCONS_00102859, MyoD, Mef2c, MyoG, MSTN and GAPDH are listed in Table S11. The qPCR reaction included one cycle of 95 °C for 2 min, followed by forty cycles of 95 °C for 5 s and 60 °C for 30 s. Melting curves were analyzed to test the accuracy of the data. The results were used for the analysis of statistically significant differences in expression.

## 3. Results

### 3.1. Overview of RNA Sequencing

We constructed eight cDNA libraries (T1–4 and U1–4) from ovine gastrocnemius muscle samples at four stages. After filtering out adaptors and low-quality reads, 182,434,881 clean reads were obtained (Table S1). Approximately 90% (89.0–91.7%) of the total clean reads were mapped to the ovine reference genome (Table S1) and a total of 6634 unique assembled transcripts were obtained.

### 3.2. Genomic Information of LncRNA in Sheep Skeletal Muscle

To identify lncRNAs, we developed a stringent filtering pipeline to discard transcripts without the characteristics of lncRNAs (Figure 1A). A total of 2470 transcripts remained after removing the transcripts with one or more of the following: homology to known genes, length of less than 200 nt, single exons and coverage of three or fewer reads. In the final step, our pipeline identified 2002 transcripts from an intersection of the results of CPC, CNCI and Pfam analyses (Table S4, Figure 1B), which included 210 intronic lncRNAs, 1241 intergenic lncRNAs and 551 antisense lncRNAs (Table S3).

**Figure 2:**
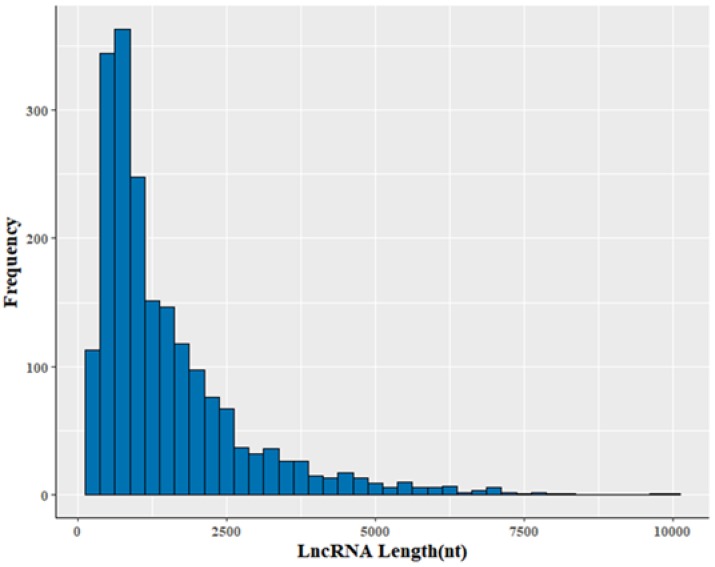
Profile of lncRNA lengths.

**Figure 3:**
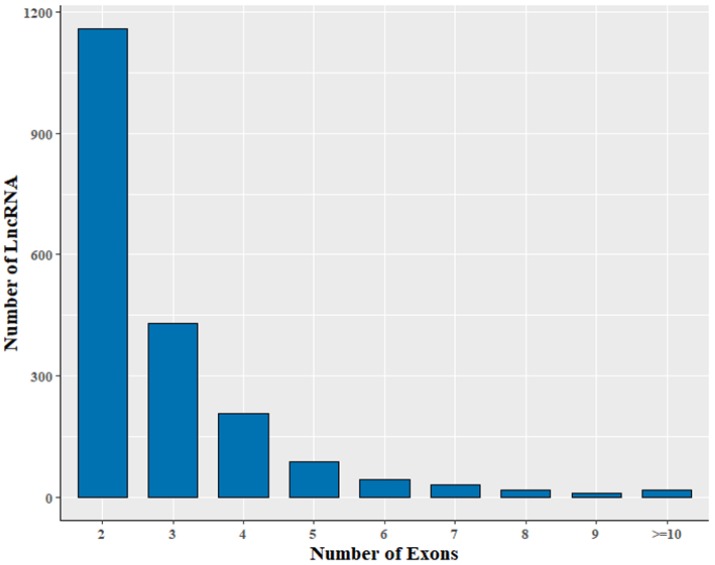
Statistics of number of lncRNA exons.

The 2002 lncRNA transcripts were distributed over all chromosomes except the Y chromosome (Figure S1). Their average length was 1510 nt, with a range of 200–9952 nt (Table S7). The size of the lncRNA transcripts was distributed mainly in the range from 200 to 1000 nt (Figure 2). Approximately 78.5% of identified lncRNA transcripts were shorter than 2000 nt. The average number of exons was 2.9. In addition, transcripts containing two exons were the most common, accounting for 57.8% of the 2002 lncRNAs (Figure 3).

### 3.3. Identification of Differentially Expressed LncRNAs

**Figure 4:**
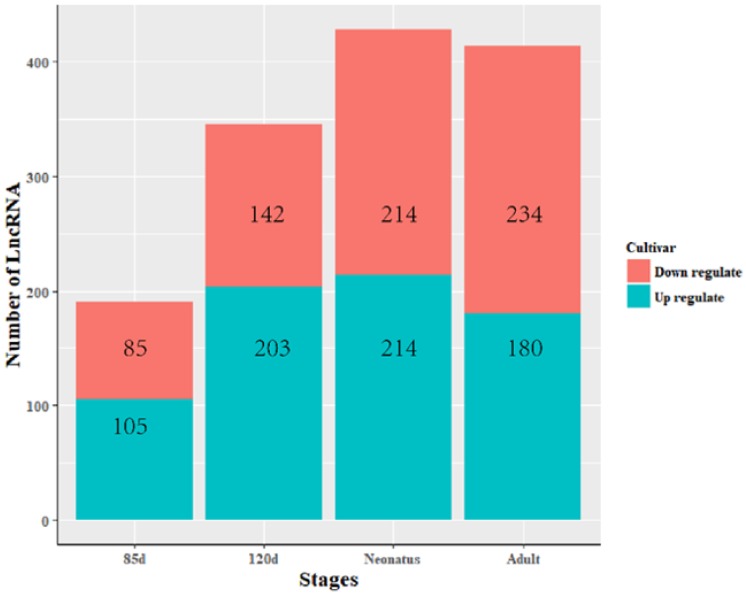
Numbers of lncRNAs differentially expressed between Texel and Ujumqin at four stages. The expression levels of lncRNA transcripts were estimated using FPKM. At the same developmental phase, the expression levels of lncRNA transcripts were compared between Texel and Ujumqin samples. Red represents down-regulated lncRNAs and blue represents up-regulated ones. On fetal day 85, 190 DEGs were found, including 85 down-regulated DEGs and 105 up-regulated DEGs. On fetal day 120, there were 345 DEGs, including 142 down-regulated DEGs and 203 up-regulated DEGs. In neonates, there were 428 DEGs, including 214 down-regulated DEGs and 214 up-regulated DEGs. Finally, in adults, there were 414 DEGs, including 234 down-regulated DEGs and 180 up-regulated DEGs. Overall, the numbers of down- and up-regulated DEGs generally increased with age.

**Figure 5:**
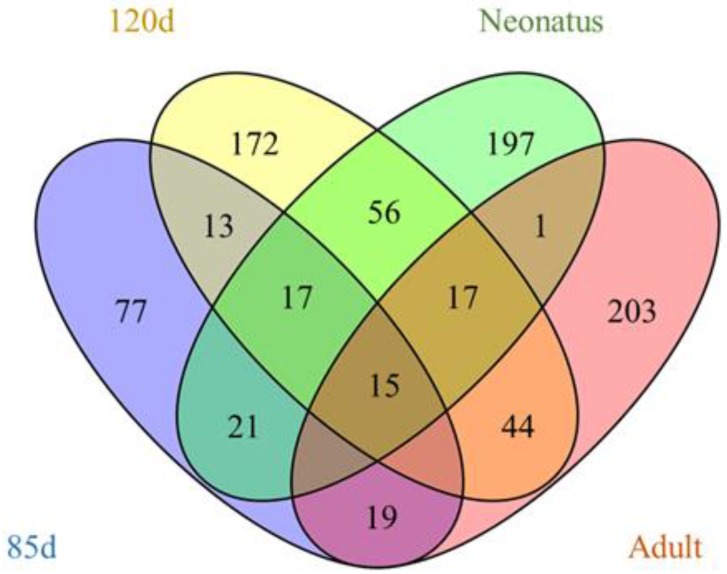
Venn diagram showing the differentially expressed lncRNAs at the four stages.

**Figure 6:**
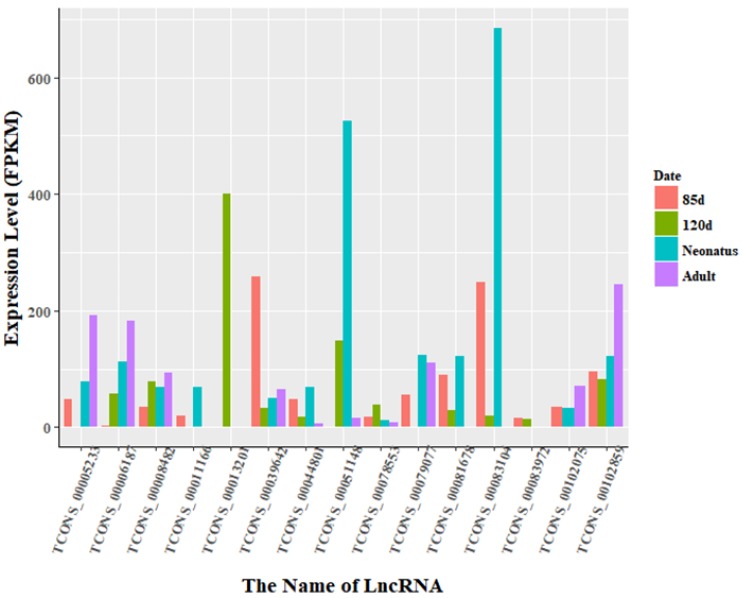
Expression level of differentially expressed lncRNAs at all stages in Texel. There were 15 lncRNAs that were identified to be differentially expressed in all stages. The expression levels of lncRNA transcripts were estimated using FPKM. In particular, the expression of *TCONS_00006187* increased gradually with development in Texel sheep. In contrast, *TCONS_00013201* was expressed only in Texel 120-day-old fetus. Moreover, the expression level of *TCONS_00083104* in Texel 85-day-old fetus and neonate was higher than at other stages. Finally, *TCONS_00044801, TCONS_00008482* and *TCONS_00102859* were expressed steadily at all four stages in Texel sheep. While the 6 lncRNAs we mentioned above show different expressed characters in Ujumqin sheep.

**Figure 7:**
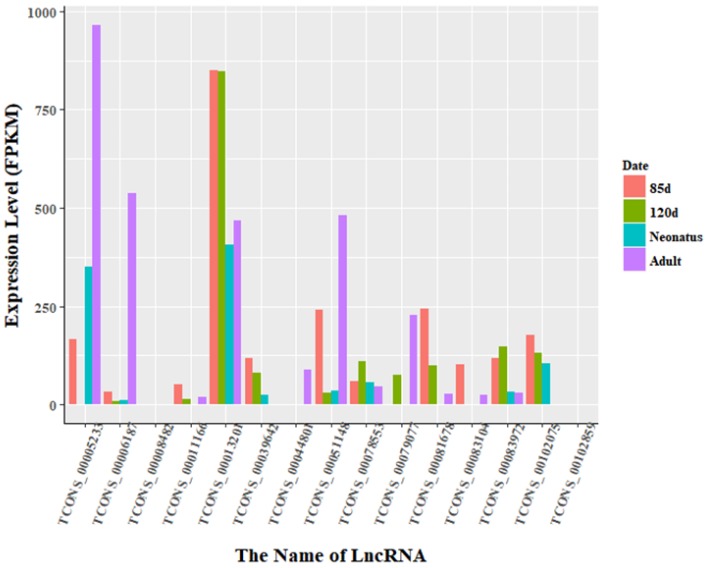
Expression level of differentially expressed lncRNAs at all stages in Ujumqin. There were 15 lncRNAs that were identified to be differentially expressed in all stages. The expression levels of lncRNA transcripts were estimated using FPKM. In particular, the expression of *TCONS_00006187* was markedly higher in adult Ujumqin sheep than at other stages. Moreover, *TCONS_00013201* was highly expressed at all stages in Ujumqin sheep, while the expression level of *TCONS_00083104* in Texel 85-day-old fetus and adult was low. *TCONS_00044801* was expressed only in Ujumqin adult, while *TCONS_00008482* and *TCONS_00102859* were not expressed at all in Ujumqin sheep. These six lncRNAs showed different expression from that in Texel sheep.

The expression levels of lncRNA transcripts were estimated using FPKM. The expression levels of lncRNA transcripts from the same developmental phase were compared between Texel and Ujumqin samples. We identified a total of 967 lncRNA transcripts that were differentially expressed in one or more of the four stages (Table S6). The number of differentially expressed lncRNAs tended to increase until parturition and then decreased thereafter. The numbers of both up- and down-regulated lncRNAs followed this same trend over the developmental stages (Figure 4). To further analyze the interactions of differentially expressed lncRNAs in the two breeds, we constructed Venn diagrams using 190, 345, 428 and 414 lncRNAs that were differentially expressed between two breeds in the samples from 85-day-old fetus, 120-day-old fetus, neonate and adult, respectively (Figure 5). We identified 15 differentially expressed lncRNA transcripts that were detected at all stages (Table S5, Figure 5). We found that *TCONS_00013201* was more highly expressed in Ujumqin. *TCONS_00006187* and *TCONS_00083104* were more highly expressed in Texel samples (Figure 6 and Figure 7). *TCONS_00044801*, *TCONS_00008482* and *TCONS_00102859* were expressed in Texel but almost completely absent from Ujumqin (Figure 6 and Figure 7).

### 3.4. Enrichment Analysis of Nearest Neighbor Genes of LncRNAs

Upon applying a cut-off of 10 kb upstream and downstream of the 2002 lncRNA transcripts, 1366 coding genes were identified as the “nearest neighbors” (Table S8). As some lncRNAs have been shown to regulate their surrounding genes [27], GO and KEGG analyses were conducted on the set of neighboring genes to provide potential information about the functions of the differentially expressed lncRNAs. These coding genes were found to participate in 40 functional categories related to cell parts, macromolecular complexes, endocytosis, enzymatic regulation, cell transduction, immune, development progress, reproductive function and so on (Figure S2). Thirty-seven pathways were identified, including those related to infectious disease, cancers, environmental adaptation, signal transduction and development (Figure S3). A second analysis, using 100 kb as the cut-off, identified 6841 coding genes close to (≤100 kb) lncRNA transcripts (Table S9), which participated in 46 functional categories (Figure S4). In addition to the categories already identified above, these genes are also involved in antioxidative reactions and virus reproduction. Thirty-eight pathways were also found near to (≤100 kb) lncRNA transcripts, which were similar to those mentioned above (Figure S5). Additionally, GO and KEGG analyses were conducted on the lncRNAs differentially expressed between Ujumqin and Texel samples at the same developmental stage. Thirty-one GO categories and eleven pathways were enriched at fetal day 85. Twenty-two GO categories and five pathways were enriched at fetal day 120. Forty-three GO categories and twenty-one pathways were enriched in neonates. Twenty-four GO categories and four pathways were enriched in adults (File S1). Moreover, four target genes were found near the 15 differentially expressed lncRNA transcripts that were common to all time points. *MYOZ2*, *FOS* and *FGF2* (and its antisense gene *NUDT6*) were found close to *TCONS_00039642*, *TCONS_00044801* and *TCONS_0007907*, respectively.

### 3.5. Overexpression of TCONS_00102859 in Skeletal Muscle and Its Effects

**Figure 8:**
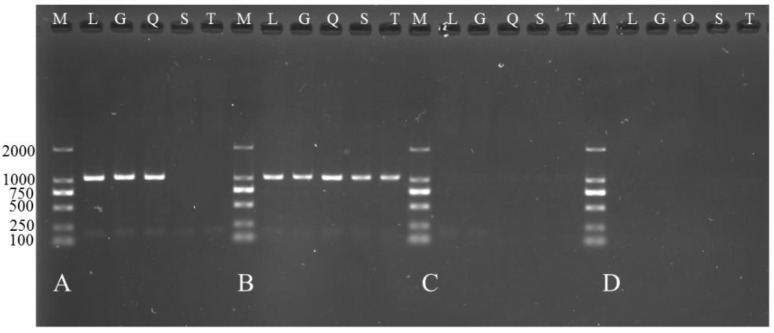
Expression of *TCONS_00102859* in skeletal muscle at different ages in Ujumqin and Texel. (A) Expression of *TCONS_00102859* in skeletal muscle of 85-day-old Texel fetus. (B) Expression of *TCONS_00102859* in skeletal muscle of Texel neonate. (C) Expression of *TCONS_00102859* in skeletal muscle of 85-day-old Ujumqin fetus. (D) Expression of *TCONS_00102859* in skeletal muscle of Ujumqin neonate. From left to right, the samples are markers (M), longissimus dorsi (L), gastrocnemius muscle (G), quadriceps femoris (Q), scapula muscle (S) and triceps (T).

**Figure 9:**
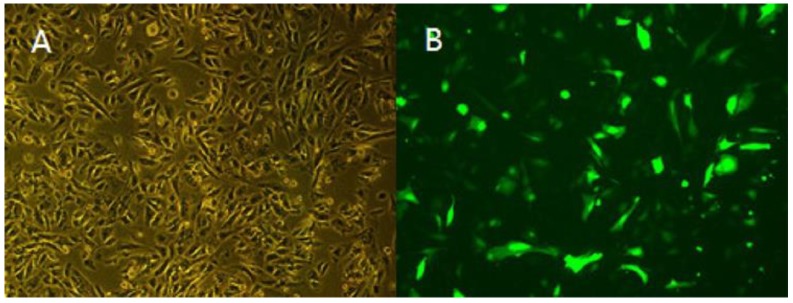

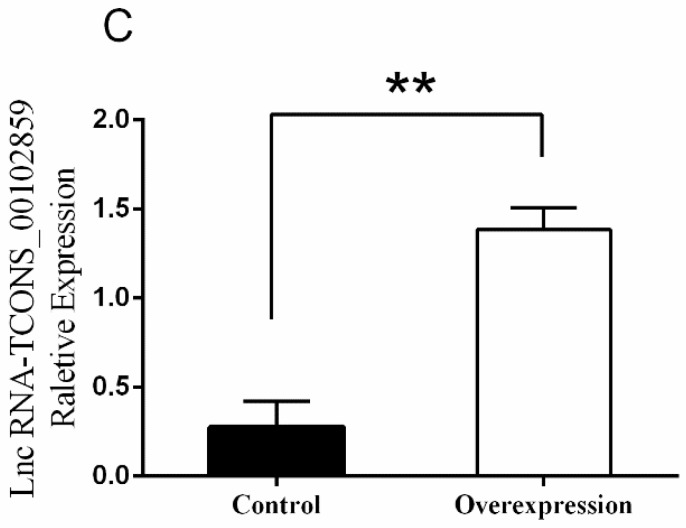
Fluorescence analysis of myoblasts cells transfected with lentiviral vectors (10×). (**A**) Positive cells were observed using a light microscope (10×). (**B**) Positive cells were observed using a fluorescence microscope (10×). (**C**) Expression level of *TCONS_00102859* in myoblasts. The expression level of *TCONS_00102859* in test group was significantly higher than that in control (*p* < 0.01). ‘*’ represents significant difference (*p* < 0.05) and ‘**’ represents extremely significant difference (*p* < 0.01).

**Figure 10:**
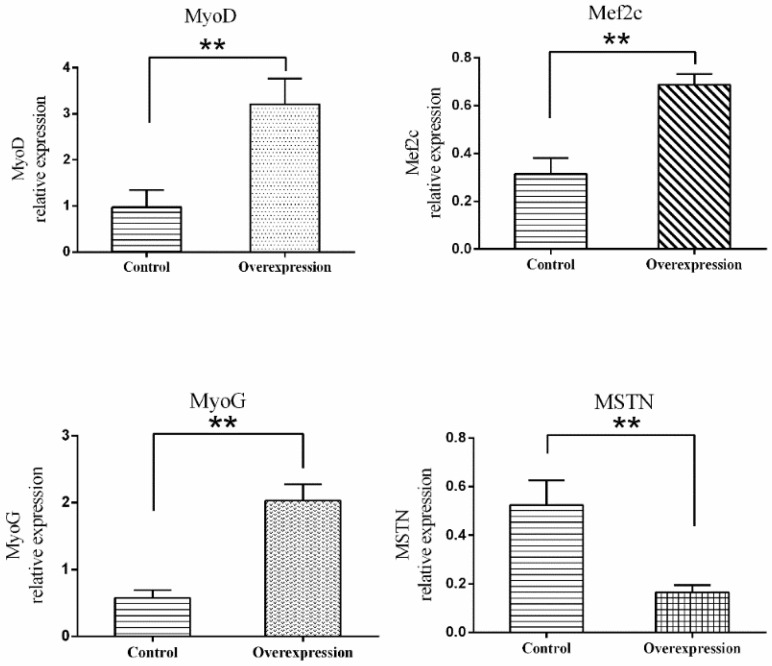
Expression levels of *MyoD, MyoG, Mef2c* and *MSTN* in myoblasts transfected with GV287-TCONS_00102859. To investigate the influence of the overexpression of *TCONS_00102859* on myoblasts, GV287-TCONS_00102859, for which stable expression can be achieved, was used. Cells transfected with GV287-N empty vector were set as a control. We detected four genes related to muscle development. Among these, the expression levels of *MyoD, MyoG* and *Mef2c*, which had a positive impact on myogenesis, were significantly higher than those of the control (*p* < 0.01). Meanwhile, the expression level of *MSTN*, which can inhibit myogenesis, was significantly lower than that of the control (*p* < 0.01). ‘*’ represents significant difference (*p* < 0.05) and ‘**’ represents extremely significant difference (*p* < 0.01).

The results of RT-PCR on various muscle indicated that TCONS_00102859 was expressed in hindlimb skeletal muscle of 85-day-old Texel fetus and forelimb and hindlimb skeletal muscle of Texel neonate. In contrast, there was no expression in Ujumqin (Figure 8). These findings were in accordance with the result of RNA-seq. 72 h after transfection, about 70% cells expressed the GFP reporter gene (Figure 9), which suggested that the target gene had been integrated successfully into chromosomes. Besides, the qPCR result of *TCONS_00102859* showed it expressed steadily and significantly higher in test group (*p* < 0.01) (Figure 9C). In transfected cells, the expression levels of *MyoD, MyoG* and *Mef2c*, which have positive impacts on myogenesis, were significantly higher than those in the control (*p* < 0.01) (Figure 10). Meanwhile, the expression level of *MSTN* which can inhibit myogenesis was significantly lower than that of the control (*p* < 0.01) (Figure 10).

## 4. Discussion

LncRNA sequencing of eight RNA libraries was conducted by Illumina HiSeq. After trimming and screening out low-quality reads, 182,434,881 reads were obtained. Subsequently, Cufflinks was used to assemble the reads that were aligned to the ovine reference genome (Oar_v3.1). After alignment with the reference genome, 5162 gene models and 6634 potential gene transcripts were identified. We calculated the coding potential of these predicted transcripts and found 780 novel coding transcripts and 5854 noncoding transcripts. Further efforts were made to refine the group of noncoding transcripts by removing rRNA and tRNA encoding genes. Finally, 2002 lncRNA transcripts were identified. Only 35 of these were known to us. However, a number of lncRNAs can encode some proteins. Hence, there are some lncRNAs that we cannot identify using the strict cutoffs based on lack of coding potential.

Because ncRNA usually emanated from the complex transcriptional loci, where the ncRNA was always transcribed with its associated protein coding transcript [30], analysis of the genomic context of these ncRNAs could help us to predict their functional role [31]. Therefore, we carried out annotation of genes near our set of lncRNAs to predict their potential target genes. Among these predicted target genes, there were six functional categories that are relevant to growth and development, namely, reproduction progress, metabolic process, catalysis, growth, development progress and cellular component biogenesis. Moreover, seven pathways were relevant to growth and development, namely, digestive system, cell growth and death, transport and catabolism, transcription and translation, metabolism of lipid, metabolism of energy and metabolism of carbohydrate. These results suggest that the genes close to the lncRNAs could play an important role in muscle development and transcriptional regulation.

In the 85 d fetus dataset, two signaling pathways—Wnt and MAKP—were enriched. The Wnt family of signaling proteins participates in multiple developmental events, including embryonic development, tissue maintenance, mitogenic stimulation, cell fate specification and differentiation [32,33], while the p38 MAKP signaling pathway plays a vital role in osteoblast differentiation [34]. This suggests that the growth and development of the fetus may be regulated and controlled by Wnt and MAKP and that lncRNAs may participate in this development. In the 120 d fetus dataset, five pathways were enriched. Three of these are related to cardiac muscle and heart function. Moreover, in new-born lamb, 43 GO terms and 21 pathways, which were implicated in the adaptability of newborns, were enriched. These results may be due to the change of environment associated with leaving the womb. After delivery, the change in environment induces biological processes that are required for adaptation to this. It appears that lncRNAs are involved in most biological events. In 1-year-old sheep, the number of pathways identified in the dataset decreased sharply. This suggests that, as lamb development reaches its end, the influence of lncRNAs recedes.

We compared the differentially expressed lncRNA transcripts identified for each of the four stages. 15 lncRNA transcripts were found to be common to all four datasets (Table S10). However, there were only 12 transcripts for which we have annotated protein-coding genes in the surrounding genomic region. These transcripts may particularly impact on the developmental process. Among these lncRNA transcripts, *TCONS_00039642*, *TCONS_00044801* and *TCONS_00079077* were found to be located close to genes that are associated with muscle development, namely, *MYOZ2*, *FOS*, *FGF2* and *NUDT6.*

Myozenin 2 (*MYOZ2*) belongs to the family of calcineurin-interacting proteins known as calsarcin or myozenin [35]. It is highly expressed in skeletal muscle and heart [35,36,37]. In skeletal muscle, its expression could inhibit the activity of calcineurin, which plays an important role in myocyte differentiation and conversion to the slow-muscle phenotype [38,39,40,41]. In other words, *MYOZ2* restricts muscle growth and development indirectly. Although the influence of *MYOZ2* on cardiac hypertrophy has been studied in humans [42,43,44], few studies have been carried out on it in sheep.

*Fos* is a family of transcription factors that includes *c-fos*, *FosB*, *Fra-1* and *Fra-2*. They encode leucine zipper proteins to dimerize with proteins of the *JUN* family, forming the transcription factor complex AP-1, which is involved in cell proliferation [45], differentiation [46] and transformation [47]. Therefore, members of the *Fos* family can affect cell processes indirectly. *C-fos* also exhibits strong transcription in web-forming mesodermal cells, which own stage-specific high growth ability [48]. It is now known that *c-fos* regulates cell growth and development of bone tissue [49]. In general, the *fos* family plays an important role in cell proliferation, differentiation and bone development.

Fibroblast growth factor 2 (*FGF2*) is known as an important regulator that not only promotes skeletal muscle cell proliferation but also inhibits differentiation [50]. In studies by Brunetti et al., *FGF2* inhibited the transcription of myogenin, which is necessary for myotube formation during myogenesis, maintaining proliferation in skeletal muscle [51,52]. In addition, *FGF2* was proposed to be an apical ectodermal ridge-derived factor that participates in limb outgrowth and patterning [53]. In general, *FGF2* is known as a vital regulator of muscle cell proliferation and differentiation. Nudix (nucleoside diphosphate linked moiety X)-type motif 6 (*NUDT6*), *FGF2*′s antisense gene, can suppress cellular *FGF-2* expression through constitutive over expression [54].

All genes mentioned above are associated with muscle or bone development. Intriguingly, these genes are located near lncRNA transcripts that were differentially expressed in all four stages. As lncRNAs often affect their target genes in a cis-acting manner, we can speculate that the genes *MYOZ2*, *FOS*, *FGF2* and *NUDT6* may be the target genes of the transcripts *TCONS_00039642*, *TCONS_00044801* and *TCONS_00079077*, resulting in the differences in muscle development between the Texel and Ujumqin sheep. While further research is required to support this conclusion, our study provides clear new directions for the analysis of lncRNA function in sheep.

In this study, we verified the function of *TCONS_00102859* in myoblasts. We determined that more than 70% of myoblasts were transfected successfully. According to the results of qPCR, we found that the overexpression of *TCONS_00102859* could up-regulate the expression of *MyoD*, *MyoG* and *Mef2c* and down-regulate the expression of MSTN. Myogenic regulatory factors (*MRFs*) family include *MyoD*, *MyoG*, *Myf5* and *MRF4*. Based on previous researches, *MyoD* and *MyoG* can transcriptional activate skeletal muscle gene expression and induce the myogenic program [55]. The induction of myogenesis by myocyte enhancer factor-2c (*Mef2c*) depends on *MyoD* and *MyoG. Mef2c* cooperates with the *MyoD* family of basic helix-loop-helix *(bHLH*) transcription factors to drive skeletal muscle development during embryogenesis; moreover, the loss of *Mef2c* in skeletal muscle results in improper sarcomere organization [56]. Some studies have demonstrated that *MSTN* decreased myoblast proliferation and fusion, changed the expression of myogenic regulatory factors [57] and inhibited myoblasts differentiation by down-regulating *MyoD* expression [58]. Our results indicated that *TCONS_00102859* could regulate skeletal muscle development by mediating the expression of *MyoD*, *MyoG*, *Mef2c* and *MSTN* directly or indirectly, which is in accordance with previous research. These results provided evidence for the role of lncRNA in skeletal muscle development in sheep, which help us to explain the mechanism of regulation by lncRNAs.

In our experiment, we found that *TCONS _00039642*, *TCONS_00044801* and *TCONS_00079077* were up-regulated in Texel samples during the interim of gestation. On days 85 to 100 after gestation, these difference between Ujumqin and Texel skeletal muscle was significant [59]. The findings in our study suggest that the development of skeletal muscle during pregnancy may be related to the expression level of *TCONS_00039642*, *TCONS_00044801* and *TCONS_00079077* by acting on the nearby genes *MYOZ2*, *FOS*, *FGF2* and *NUDT6* which are related to muscle development. In addition, although there are no genes related to muscle development near the five lncRNA transcripts (*TCONS_00008482*, *TCONS_00102859*, *TCONS_00013201*, *TCONS_00006187* and *TCONS_00083104*) which particularly expressed between Texel and Ujumqin, whether these lncRNA transcripts regulate target genes in a trans-acting manner is unknown. The functional analysis of *TCONS_00102859* provides a foundation and useful resources for future investigations of lncRNA. All findings in this study may contribute to the understanding of the regulatory functions of lncRNAs in ovine muscle development and also potentially in other mammals.

## 5. Conclusions

Domestic animals have mainly been raised for meat production. So it is of vital interest to reveal the molecular mechanisms underneath skeletal muscle formation and development. We identified and characterized ovine lncRNAs that may be involved in skeletal muscle development in Texel and Ujumqin. Our results provide a new perspective to explain the difference in muscle development of Texel and Ujumqin, for which only limited research on lncRNAs has been performed. Our results reveal that *TCONS_00044801*, *TCONS_00008482* and *TCONS_00102859* may participate in muscle development. In future studies, we plan to investigate the functions of these identified lncRNAs to provide more information that should add to our understanding of the regulatory mechanisms associated with sheep muscle development at the molecular and cellular level.

## Supplementary Materials

The following are available online at http://www.mdpi.com/2076-2615/8/7/127/s1, File S1 GO and KEGG analyses of 10/100 kb closest to lncRNA transcripts at each stage; File S2 Assessment results of FastQC; Figure S1 Distribution of lncRNA transcripts on sheep chromosome; Figure S2 GO analysis of target genes in the 10 kb closest to lncRNA transcripts; Figure S3 KEGG analysis of target genes in the 10 kb closest to lncRNA transcripts; Figure S4 GO analysis of target genes in the 100 kb closest to lncRNA transcripts; Figure S5 KEGG analysis of target genes in the 100 kb closest to lncRNA transcripts; Table S1 Statistics of clean reads in 8 samples; Table S2 Statistics of TopHat2 analysis; Table S3 Cufflinks class code statistic; Table S4 Analysis results of CPC, CNCI and Pfam; Table S5 Expression level of 15 differentially expressed lncRNA transcripts at all stages; Table S6 Differentially expressed lncRNAs transcripts in different stages; Table S7 Length of 2002 lncRNA transcripts; Table S8 Total target genes in the 10 kb closest to 2002 lncRNA transcripts; Table S9 Total target genes in the 100 kb closest to 2002 lncRNA transcripts; Table S10 Target genes of 15 differentially expressed lncRNA transcripts common to all stages; Table S11 Primers information.
