# Supplementary material for: Identification and Characterization of Long Noncoding RNAs in Ovine Skeletal Muscle
Source: Animals (Basel). 2018 Jul 23;8(7):127. doi: 10.3390/ani8070127 (PMC6071021; doi:10.3390/ani8070127)
Supplement: Supplementary file 1 [file animals-08-00127-s001.zip › supporting imformation/File.2/File.2 .docx]

Assessment results of FastQC in T1


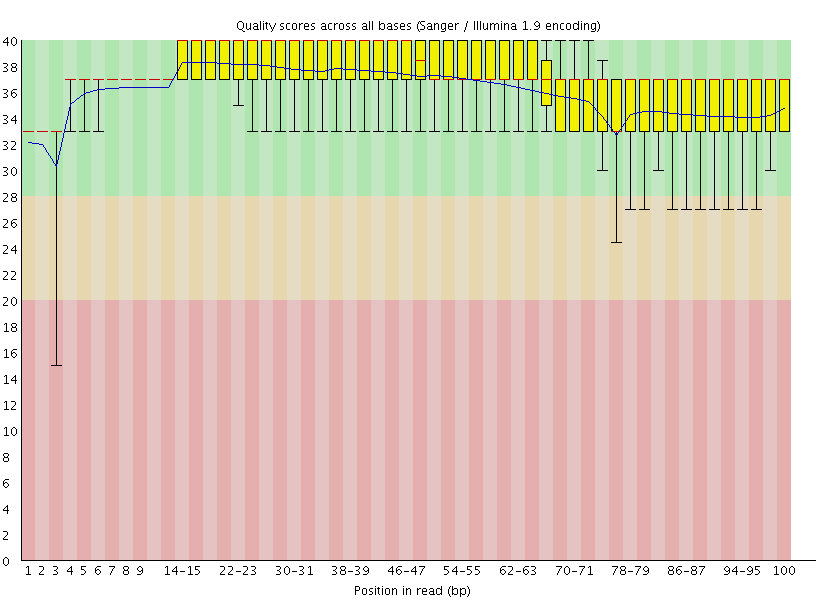


T-1.R1.per_base_quality


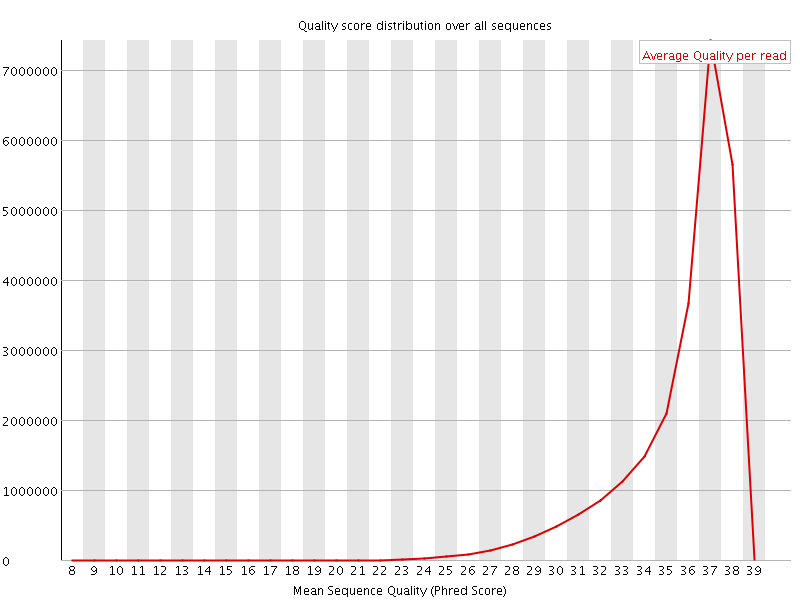


T-1.R1.per_sequence_quality


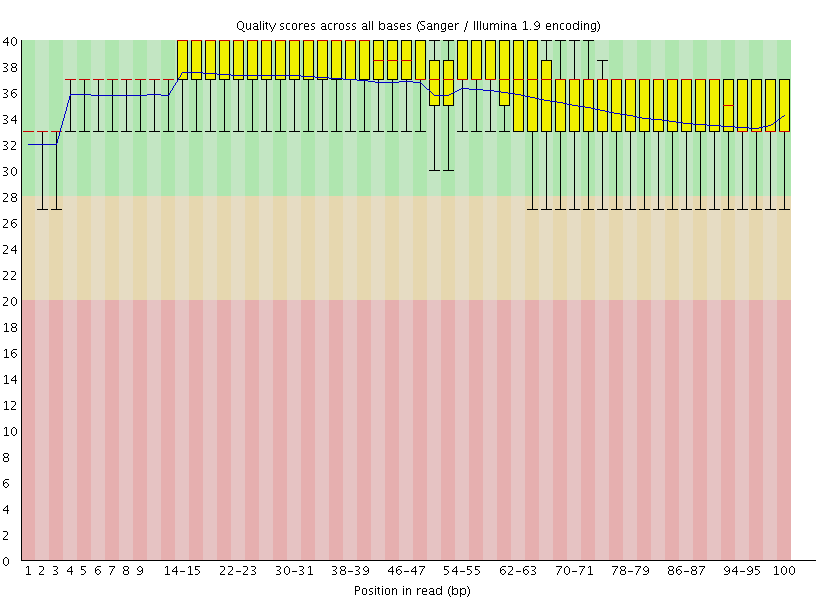


T-1.R2.per_base_quality


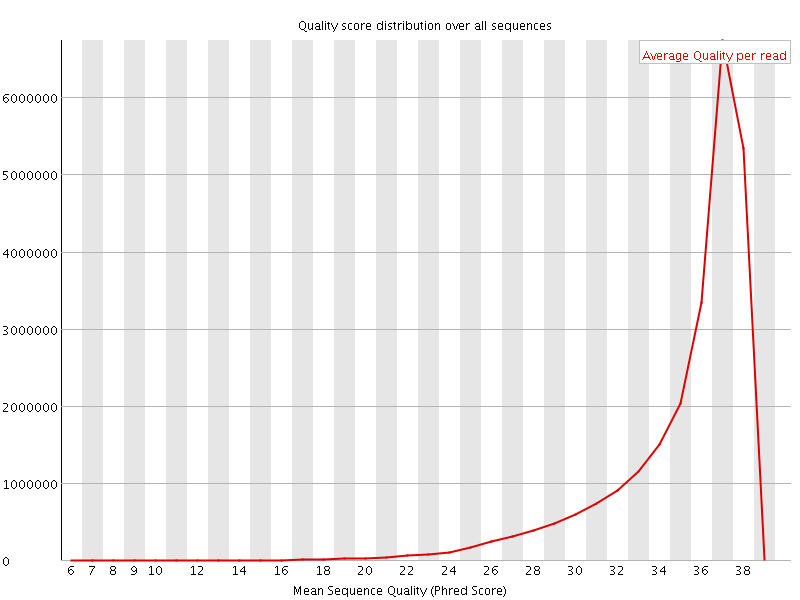


T-1.R2.per_sequence_quality

Assessment results of FastQC in T2


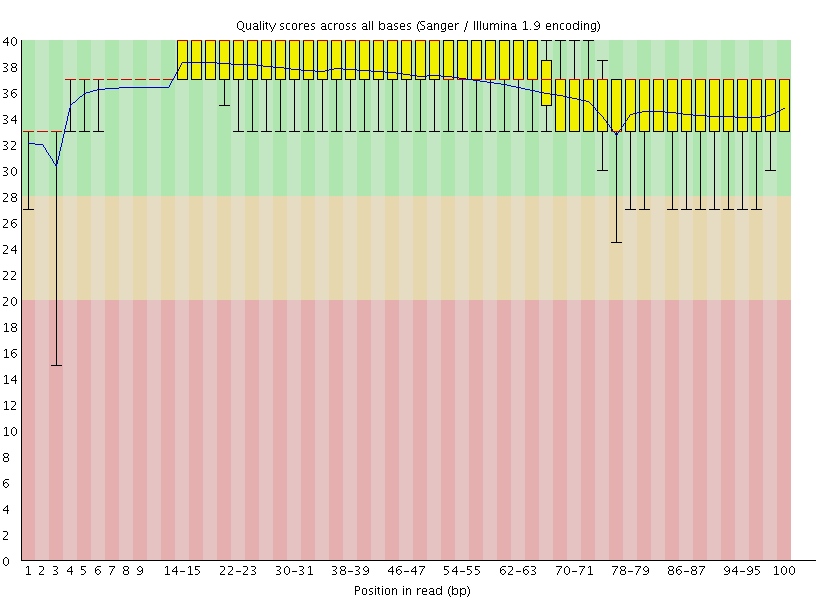


T-2.R1.per_base_quality


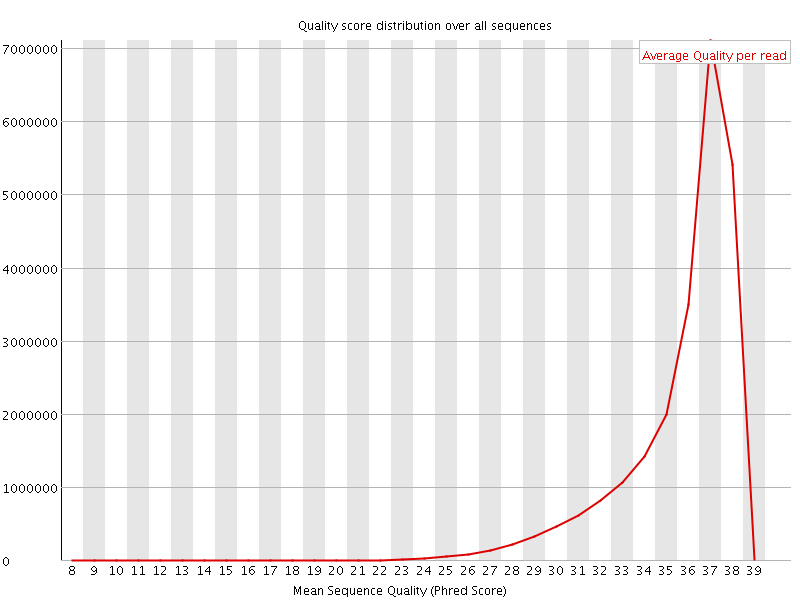


T-2.R1.per_sequence_quality


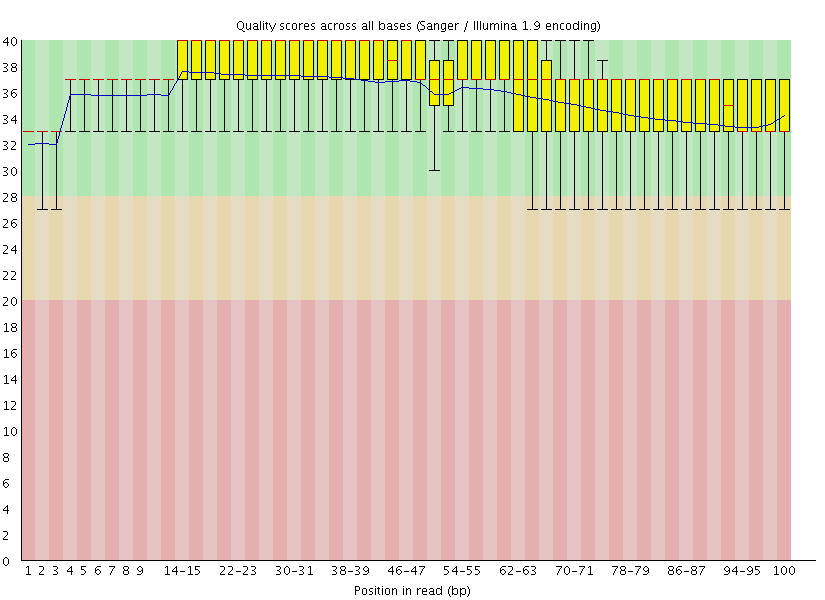


T-2.R2.per_base_quality


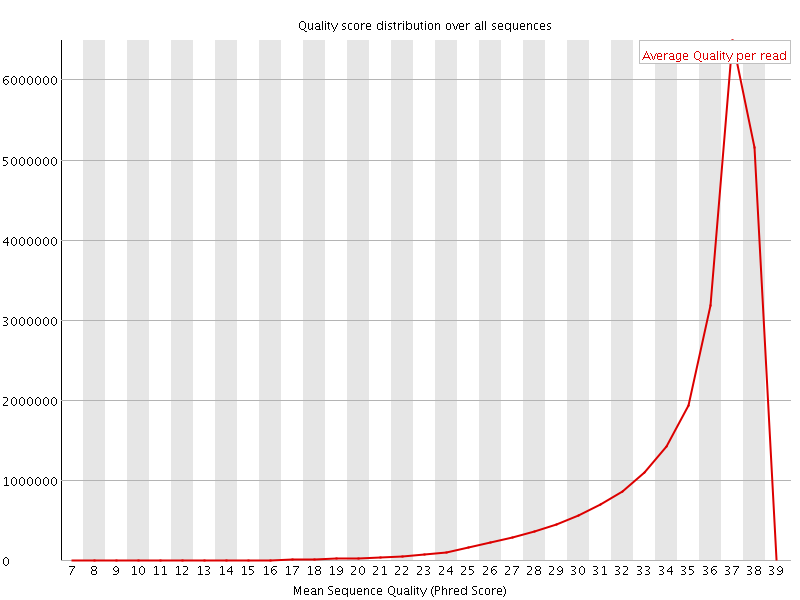


T-2.R2.per_sequence_quality

Assessment results of FastQC in T3


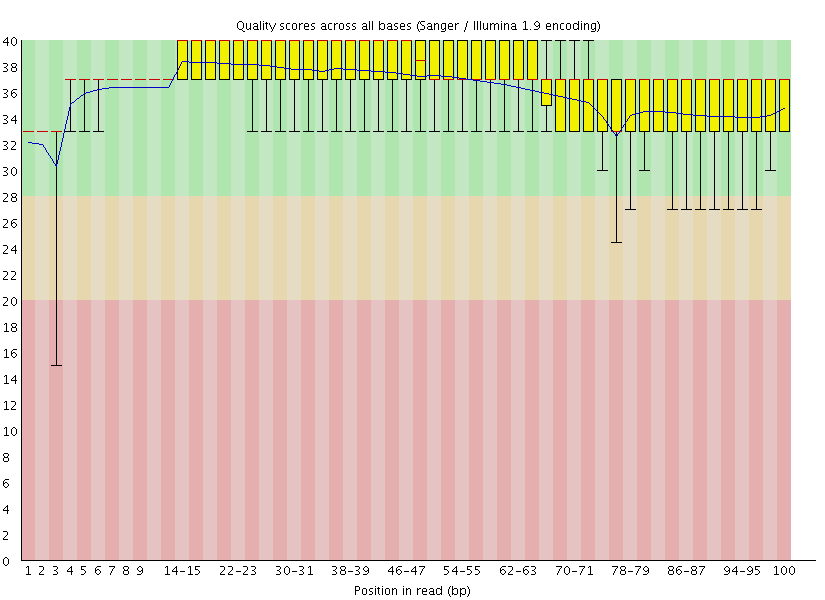


T-3.R1.per_base_quality


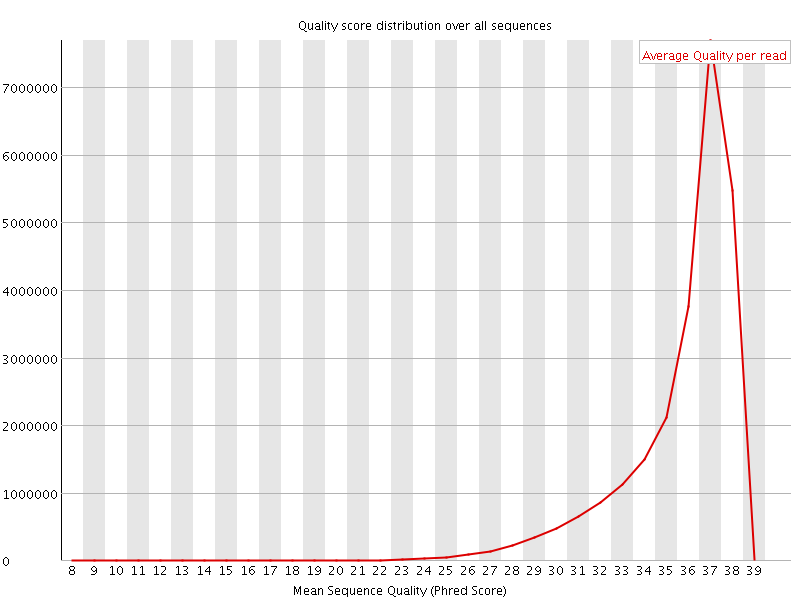


T-3.R1.per_sequence_quality


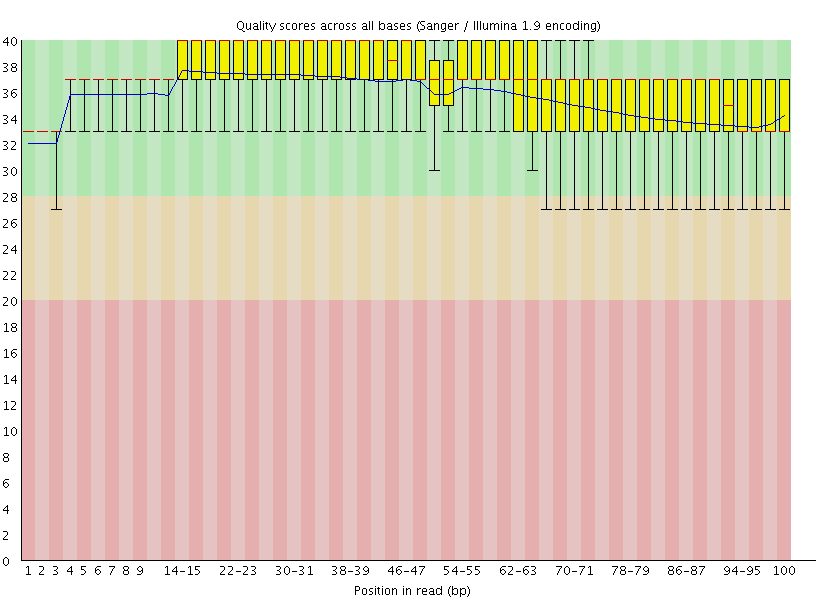


T-3.R2.per_base_quality


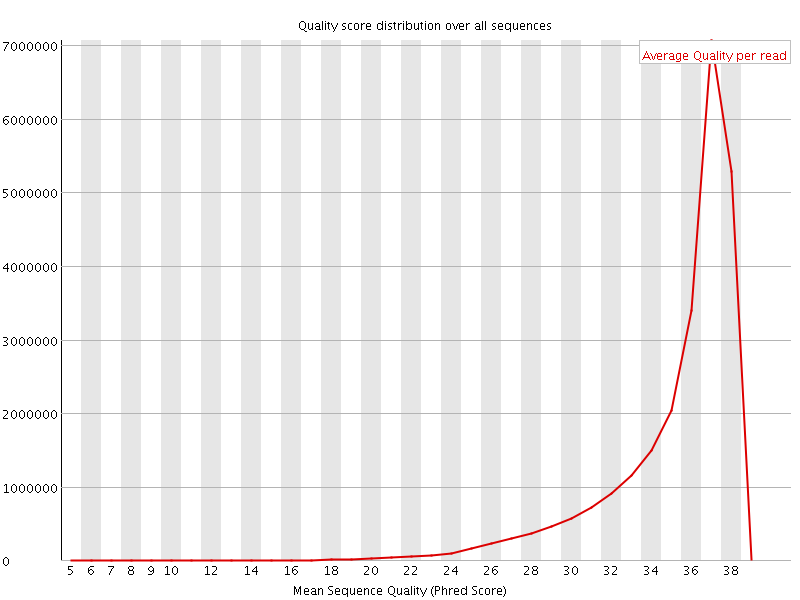


T-3.R2.per_sequence_quality

Assessment results of FastQC in T4


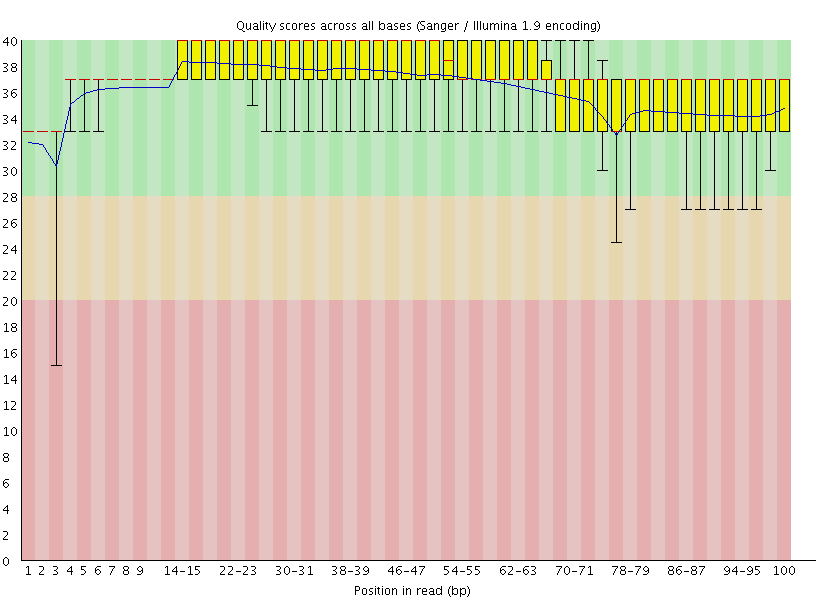


T-4.R1.per_base_quality


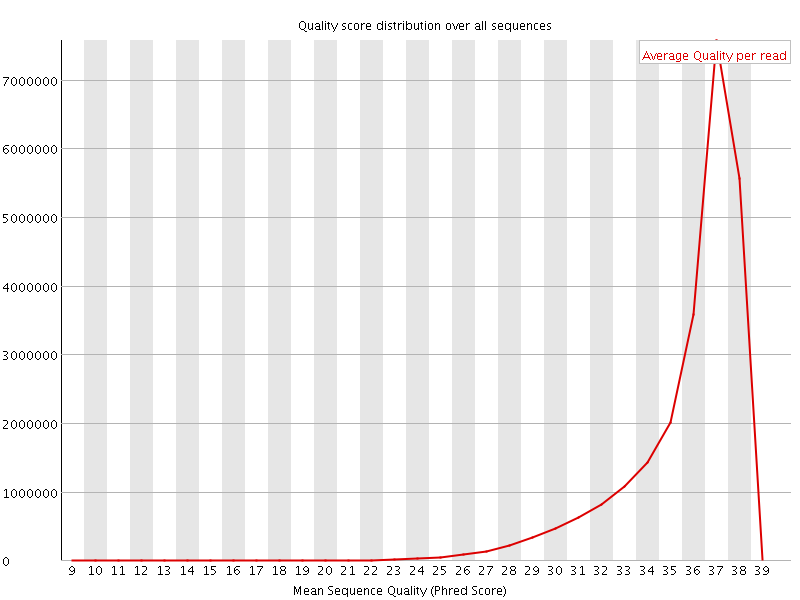


T-4.R1.per_sequence_quality


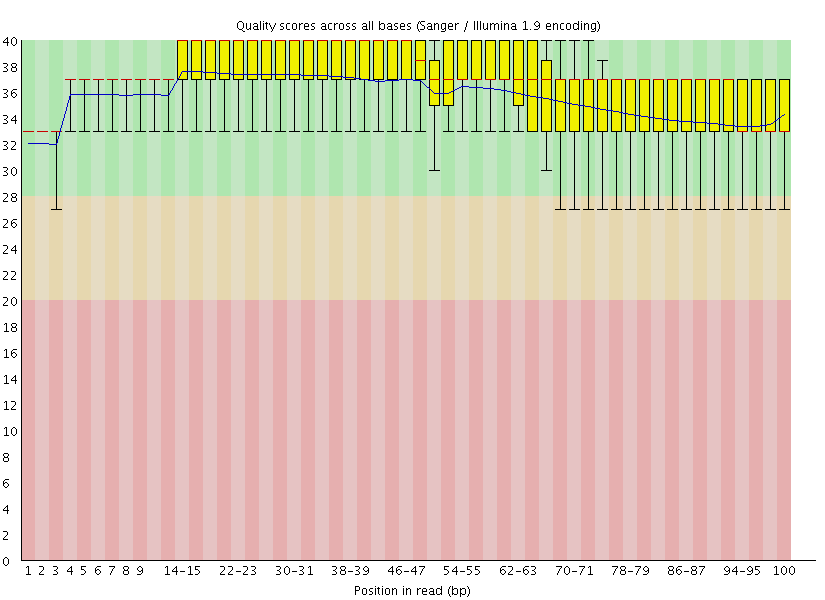


T-4.R2.per_base_quality


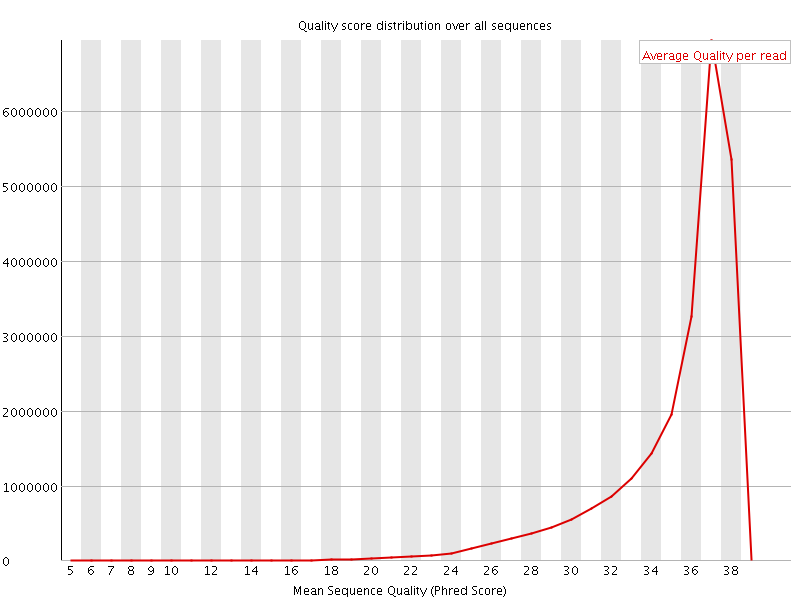


T-4.R2.per_sequence_quality

Assessment results of FastQC in U1


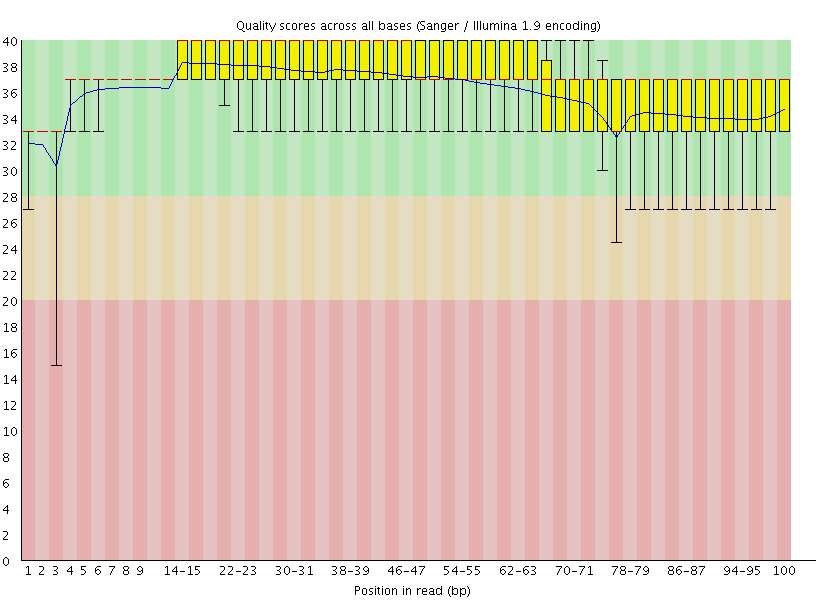


U-1.R1.per_base_quality


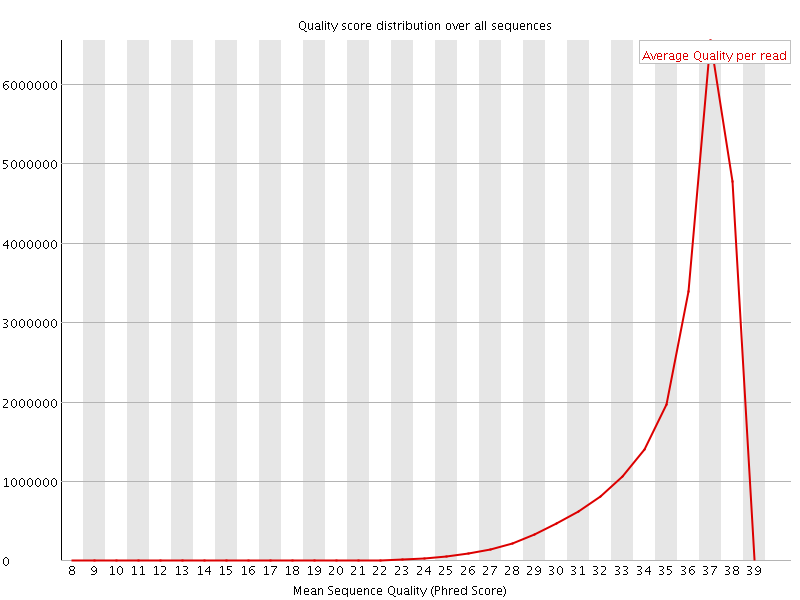


U-1.R1.per_sequence_quality


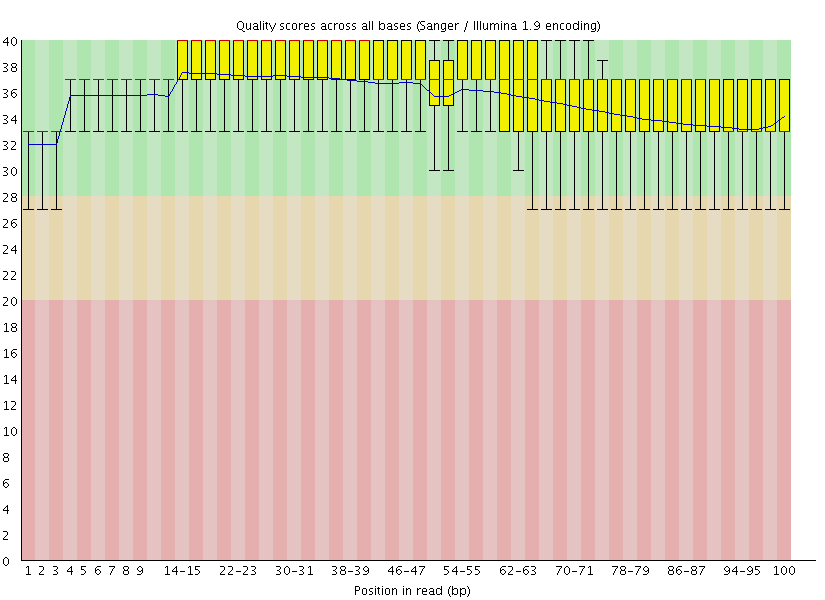


U-1.R2.per_base_quality


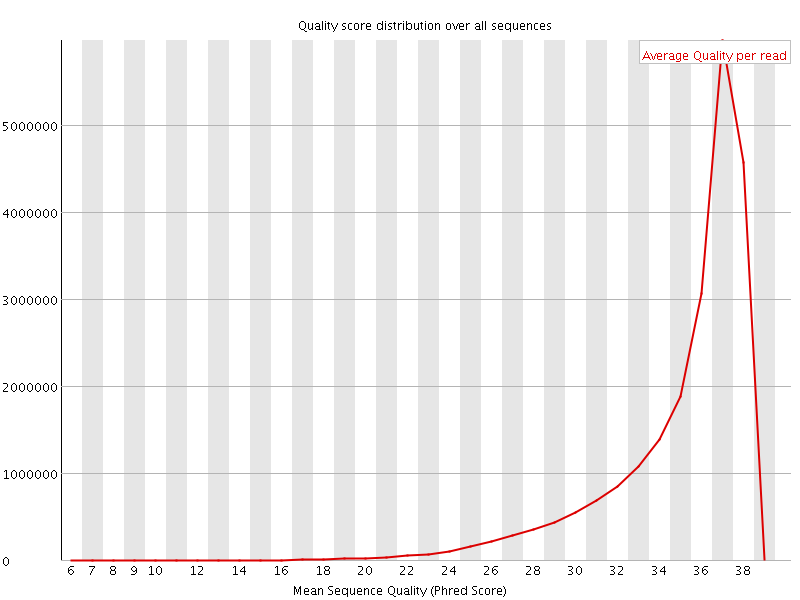


U-1.R2.per_sequence_quality

Assessment results of FastQC in U2


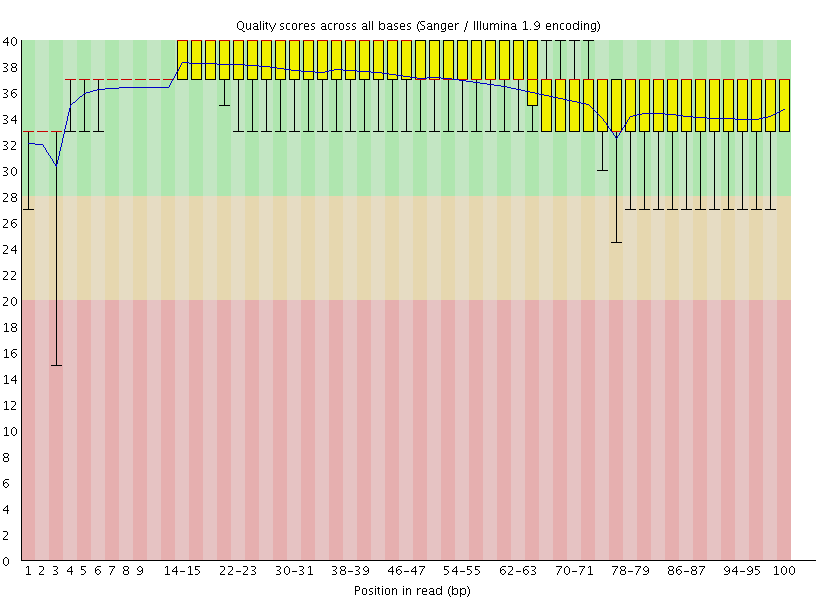


U-2.R1.per_base_quality


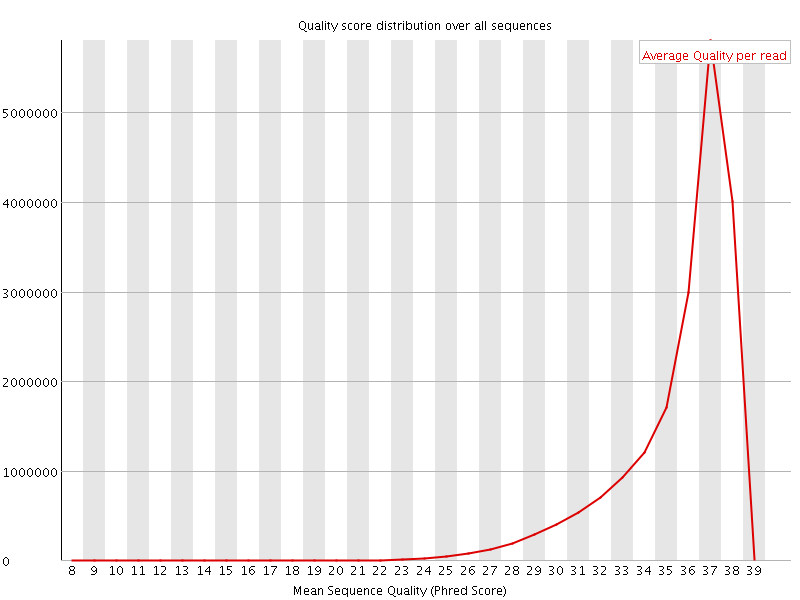


U-2.R1.per_sequence_quality


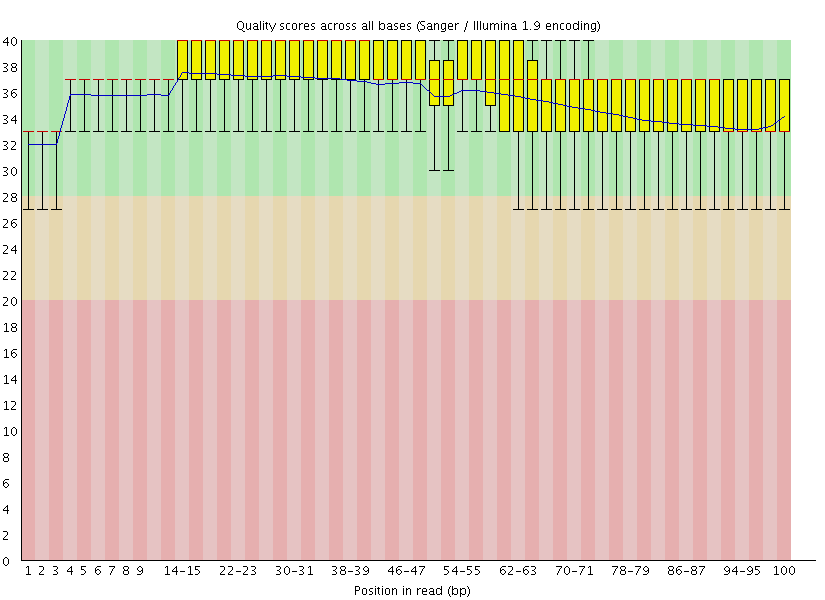


U-2.R2.per_base_quality


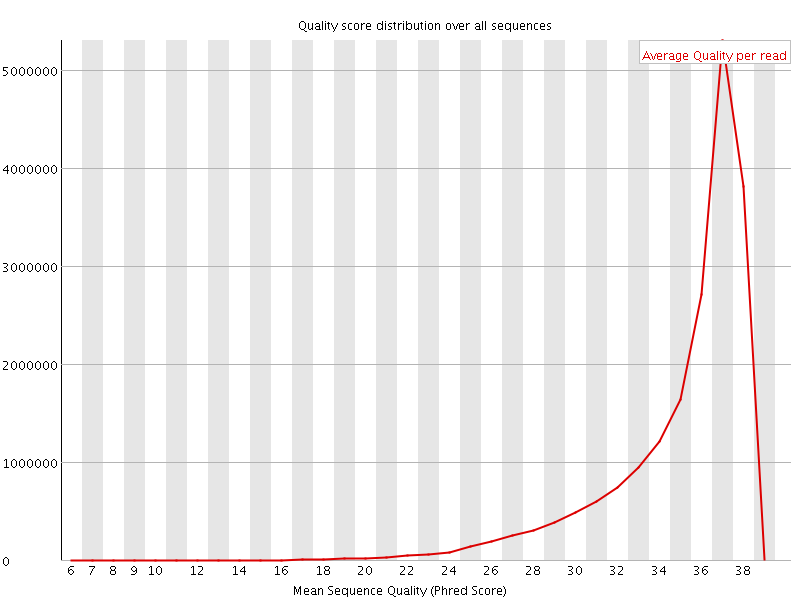


U-2.R2.per_sequence_quality

Assessment results of FastQC in U3


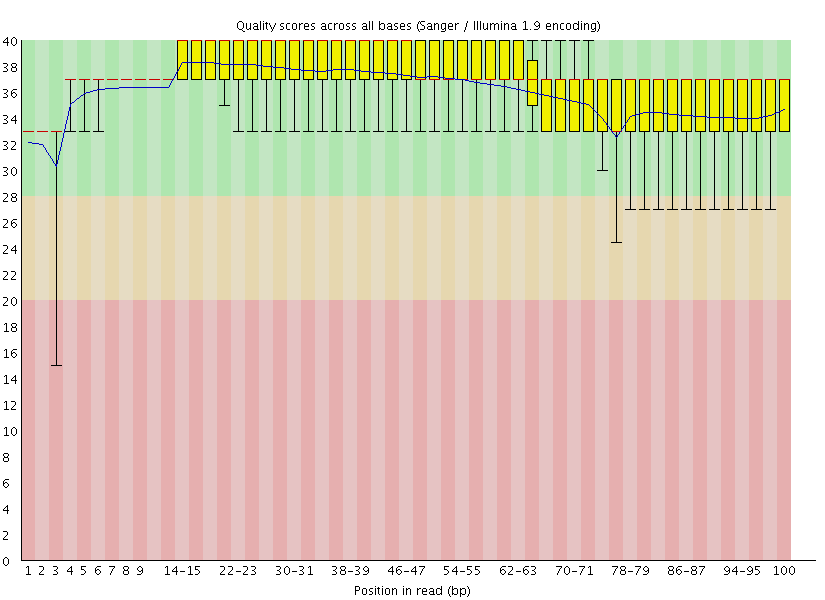


U-3.R1.per_base_quality


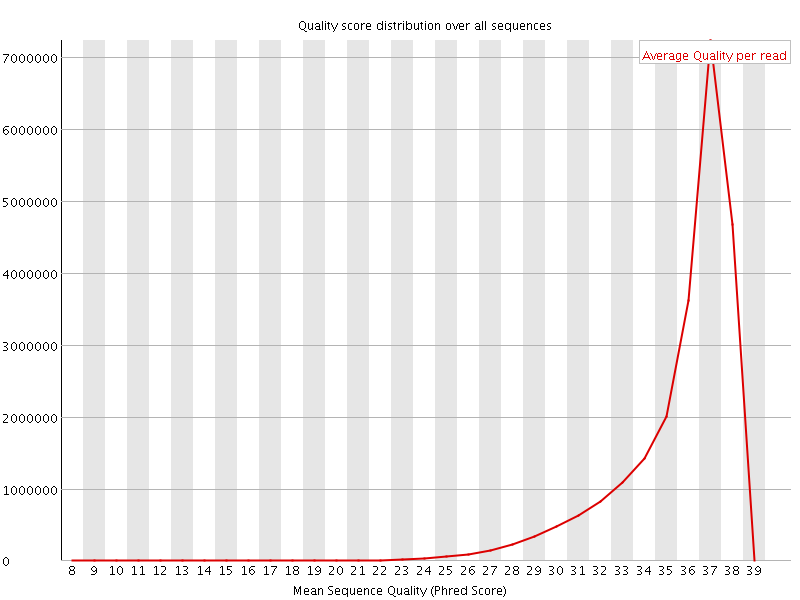


U-3.R1.per_sequence_quality


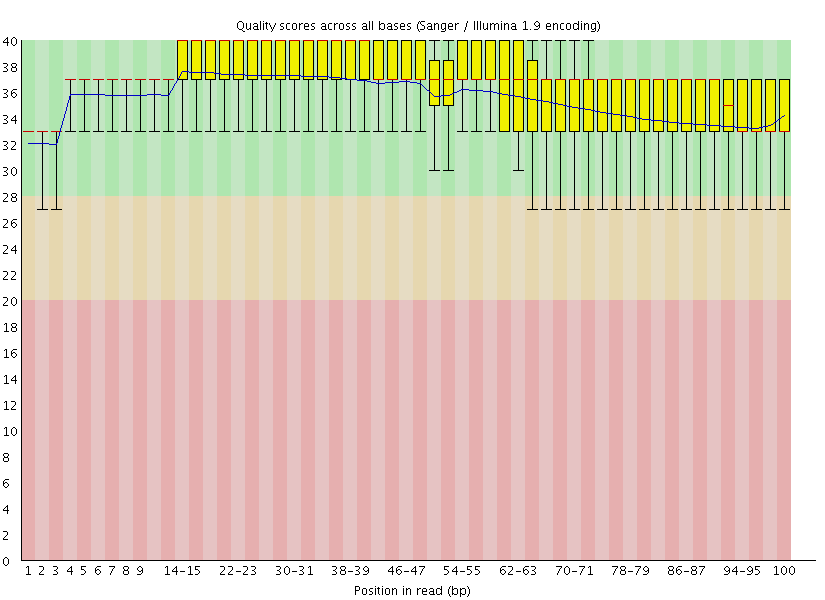


U-3.R2.per_base_quality


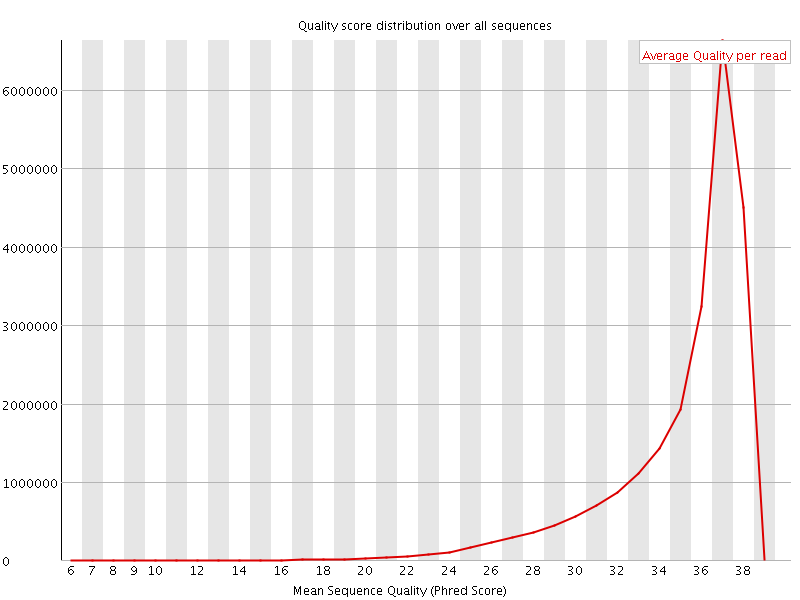


U-3.R2.per_sequence_quality

Assessment results of FastQC in U4


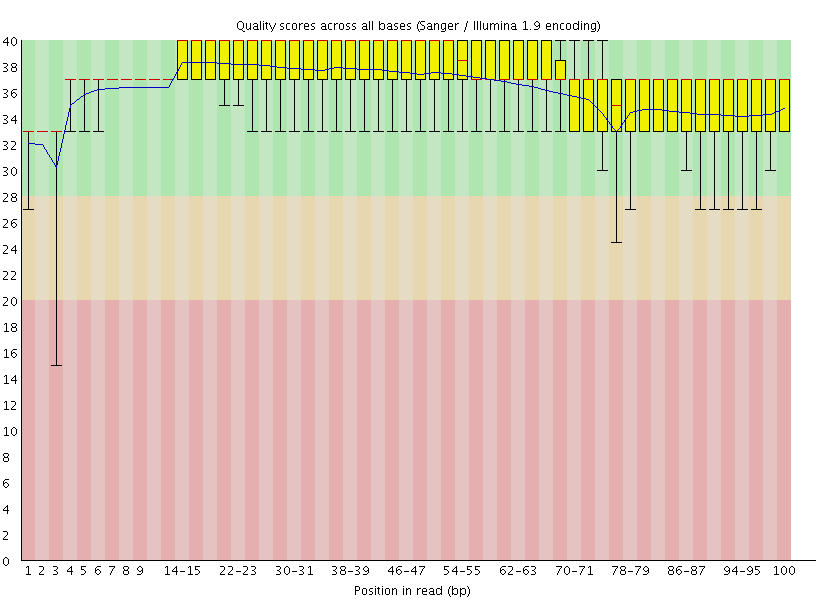


U-4.R1.per_base_quality


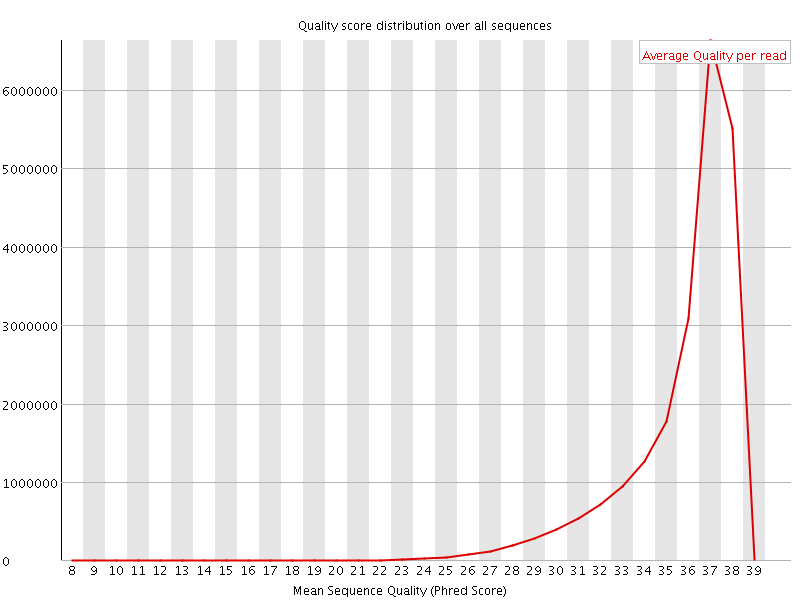


U-4.R1.per_sequence_quality


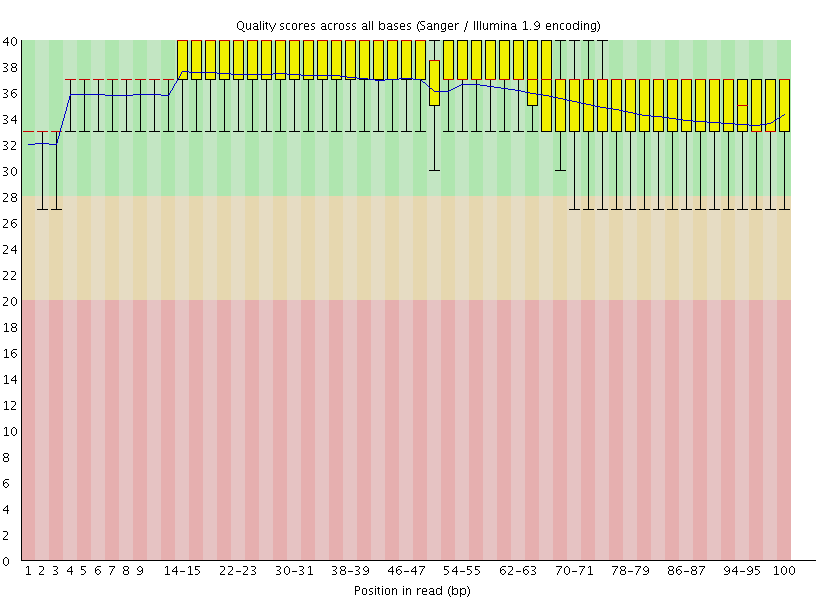


U-4.R2.per_base_quality


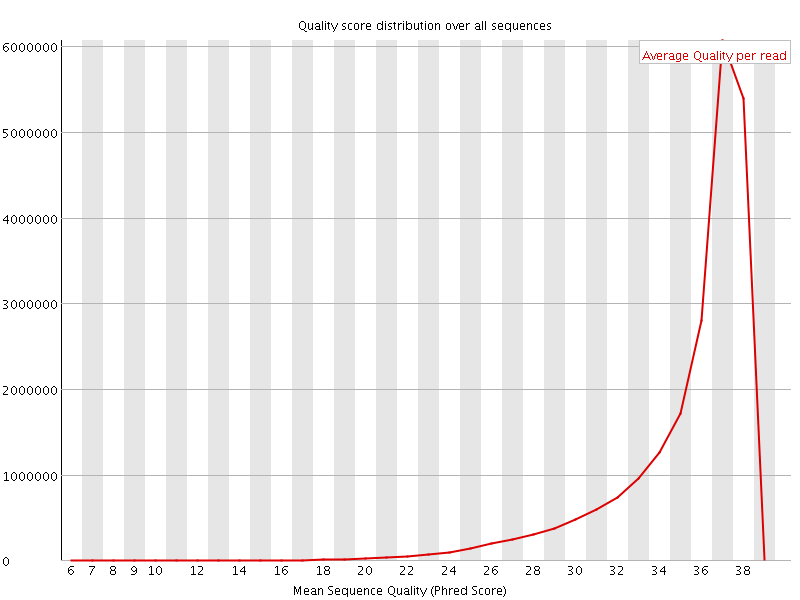


U-4.R2.per_sequence_quality
