# Supplementary material for: Identification and Characterization of Long Noncoding RNAs in Ovine Skeletal Muscle
Source: Animals (Basel). 2018 Jul 23;8(7):127. doi: 10.3390/ani8070127 (PMC6071021; doi:10.3390/ani8070127)
Supplement: Supplementary file 1 [file animals-08-00127-s001.zip › supporting imformation/Table S1.docx]

Table. S1 the statistics of clean reads in 8 samples

| sample | | Total  Sequences(bp) | Total  bases(Gbp) | Sequence  length(bp) | Q20 | GC(%) |
| --- | --- | --- | --- | --- | --- | --- |
| U1 | Reads | 21991855 | 2.2 | 20-100 | 98.3 | 49 |
| U2 | Reads | 19156070 | 1.9 | 20-100 | 98.3 | 49 |
| U3 | Reads | 22953479 | 2.3 | 20-100 | 98.0 | 50 |
| U4 | Reads | 21721760 | 2.1 | 20-100 | 98.5 | 46 |
| T1 | Reads | 24486783 | 2.4 | 20-100 | 98.2 | 49 |
| T2 | Reads | 23355710 | 2.3 | 20-100 | 98.3 | 48 |
| T3 | Reads | 24650685 | 2.4 | 20-100 | 98.0 | 49 |
| T4 | Reads | 24118539 | 2.4 | 20-100 | 98.4 | 48 |
