# Supplementary material for: Identification and Characterization of Long Noncoding RNAs in Ovine Skeletal Muscle
Source: Animals (Basel). 2018 Jul 23;8(7):127. doi: 10.3390/ani8070127 (PMC6071021; doi:10.3390/ani8070127)
Supplement: Supplementary file 1 [file animals-08-00127-s001.zip › supporting imformation/Table S2.docx]

Table S2. Statistics of TopHat2 analysis

| sample | Total pairs | Overall mapping rate(%) | Aligned Pairs | Multiple Alignments | Multiple Alignments Rate(%) | Discordant Alignments | Discordant Alignments Rate(%) | Concordant Pair Alignment Rate(%) |
| --- | --- | --- | --- | --- | --- | --- | --- | --- |
| U1 | 21991855 | 89.2 | 18300198 | 861776 | 4.7 | 322751 | 1.8 | 81.7 |
| U2 | 19156070 | 89 | 15911530 | 829113 | 5.2 | 340840 | 2.1 | 81.3 |
| U3 | 22953479 | 90.7 | 19610564 | 972177 | 5 | 201812 | 1 | 84.6 |
| U4 | 21721760 | 91.4 | 18671732 | 1302974 | 7 | 405722 | 2.2 | 84.1 |
| T1 | 24486783 | 89.9 | 20606634 | 967747 | 4.7 | 378808 | 1.8 | 82.6 |
| T2 | 23355710 | 90 | 19760078 | 1092193 | 5.5 | 298202 | 1.5 | 83.3 |
| T3 | 24650685 | 90.6 | 21046045 | 995822 | 4.7 | 320527 | 1.5 | 84.1 |
| T4 | 24118539 | 91.7 | 20907655 | 1219770 | 5.8 | 228724 | 1.1 | 85.7 |
