# Supplementary figures and images for: Identification and Characterization of Long Noncoding RNAs in Ovine Skeletal Muscle
Source: Animals (Basel). 2018 Jul 23;8(7):127. doi: 10.3390/ani8070127 (PMC6071021; doi:10.3390/ani8070127)

The distribution of lncRNA on the sheep chromosome


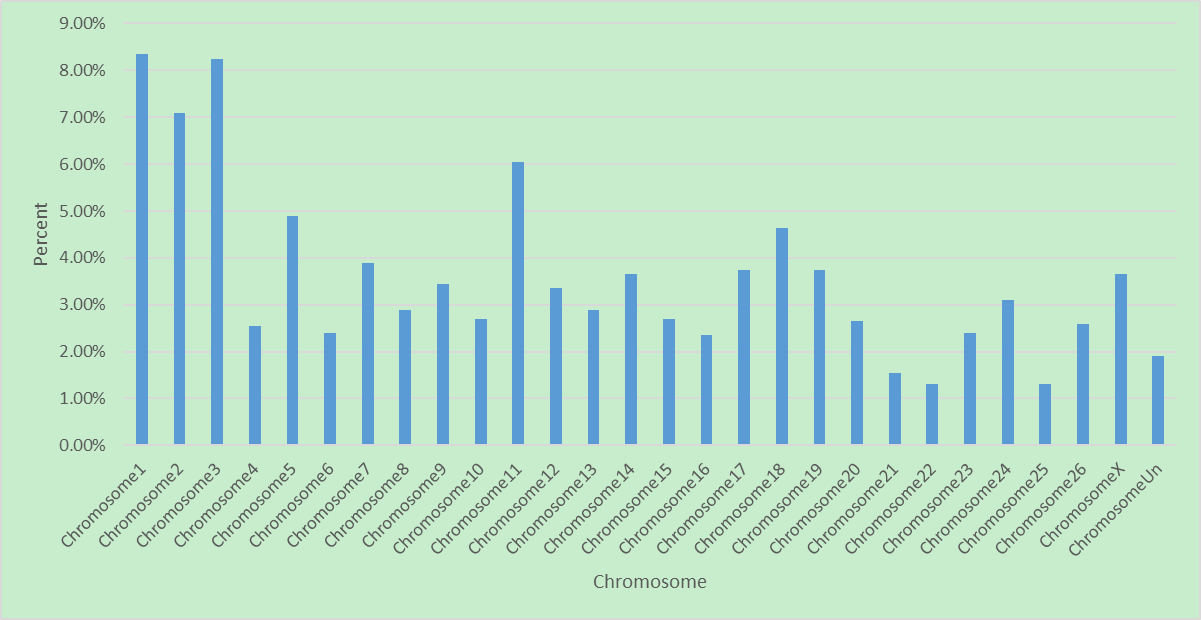

Supplement: Supplementary file 1 [file animals-08-00127-s001.zip › supporting imformation/Figure S1.docx]

GO analysis of closest 10k target gene of lncRNA


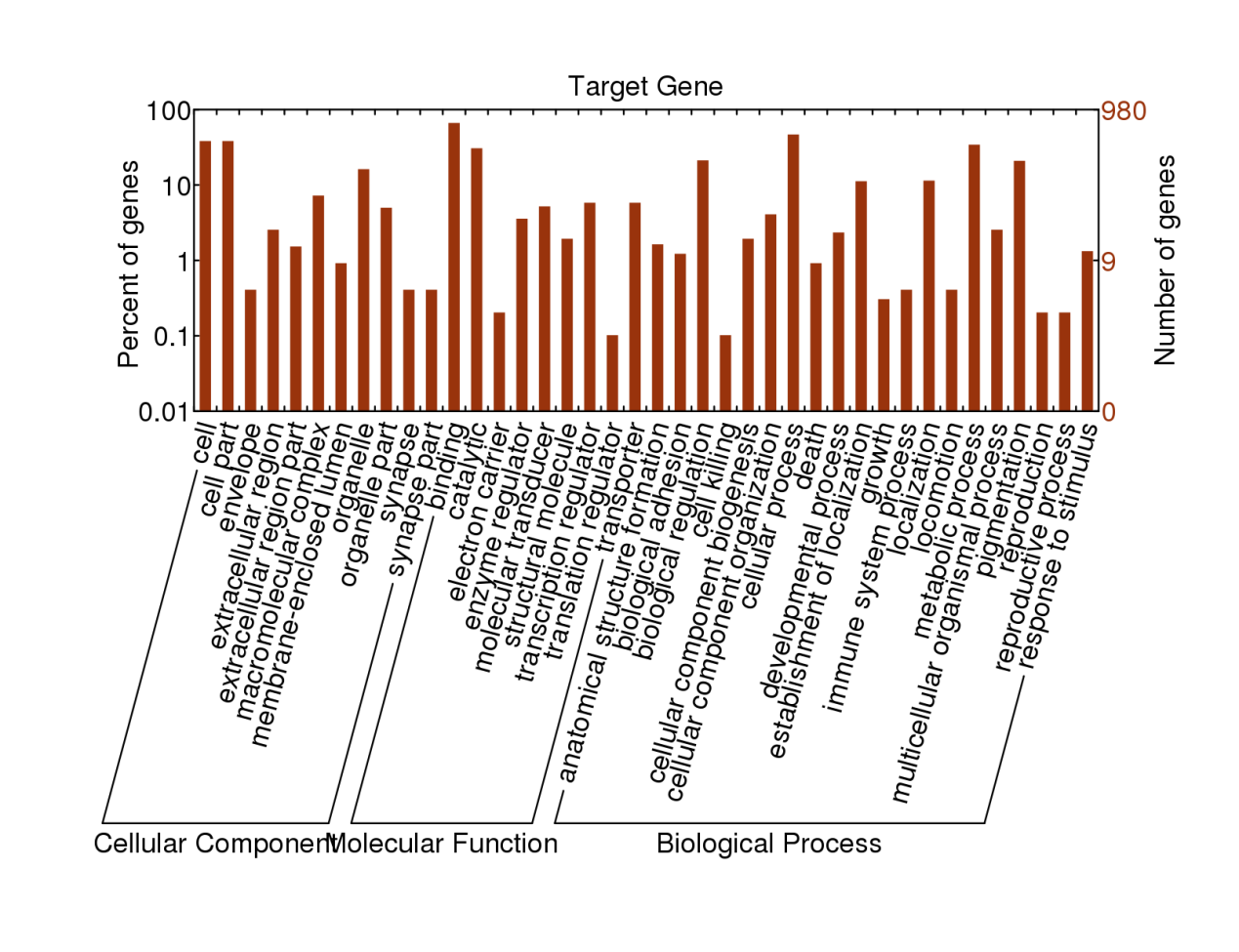

Supplement: Supplementary file 1 [file animals-08-00127-s001.zip › supporting imformation/Figure S2.docx]

KEGG analysis of closest 10k target gene of lncRNA


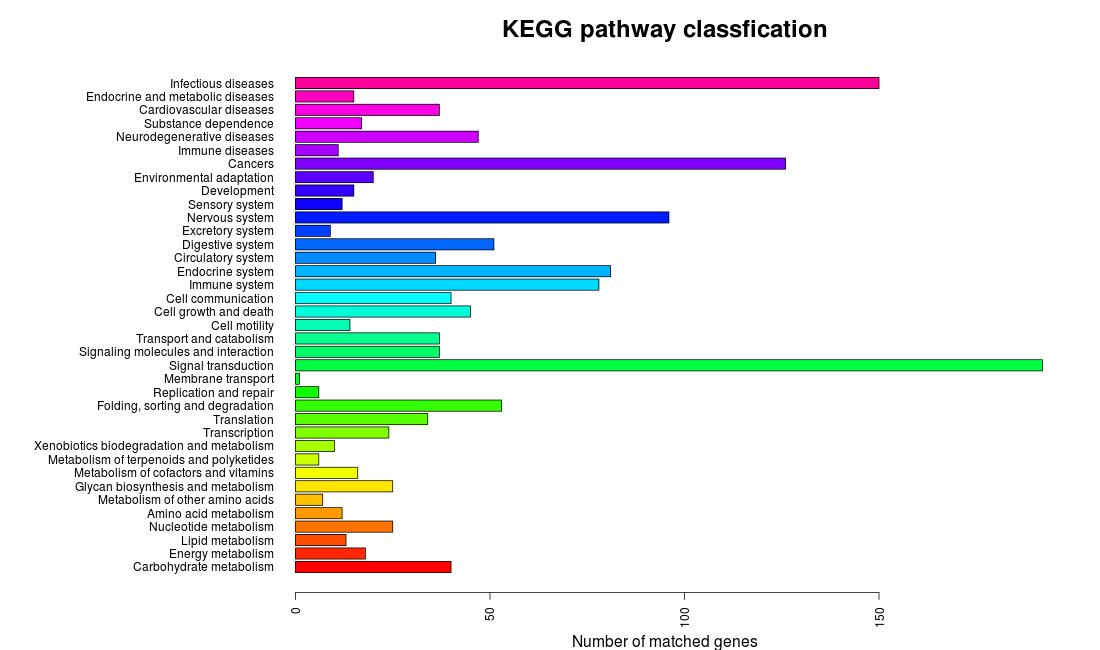

Supplement: Supplementary file 1 [file animals-08-00127-s001.zip › supporting imformation/Figure S3.docx]

GO analysis of closest 100k target gene of lncRNA
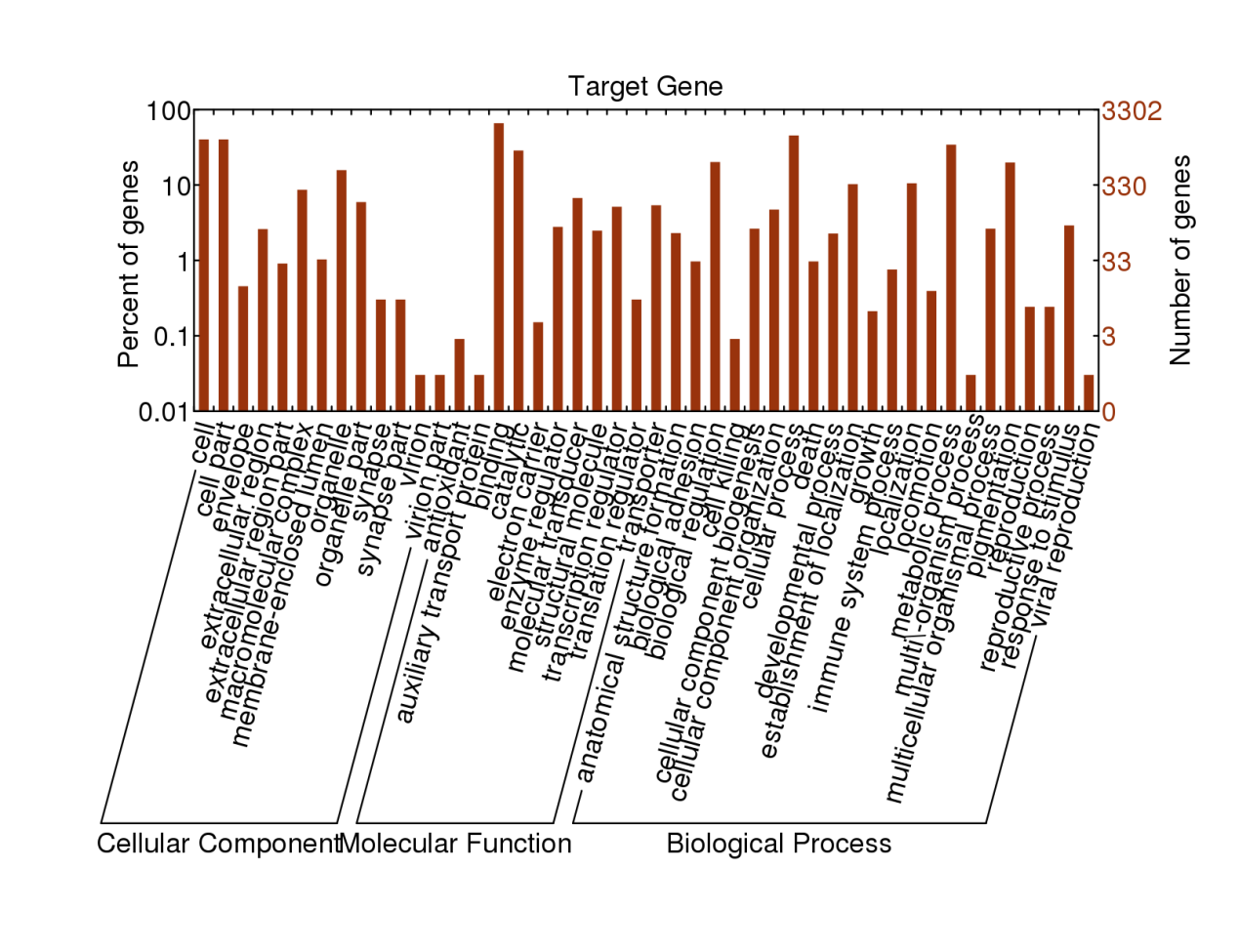

Supplement: Supplementary file 1 [file animals-08-00127-s001.zip › supporting imformation/Figure S4.docx]

KEGG analysis of closest 100k target gene of lncRNA


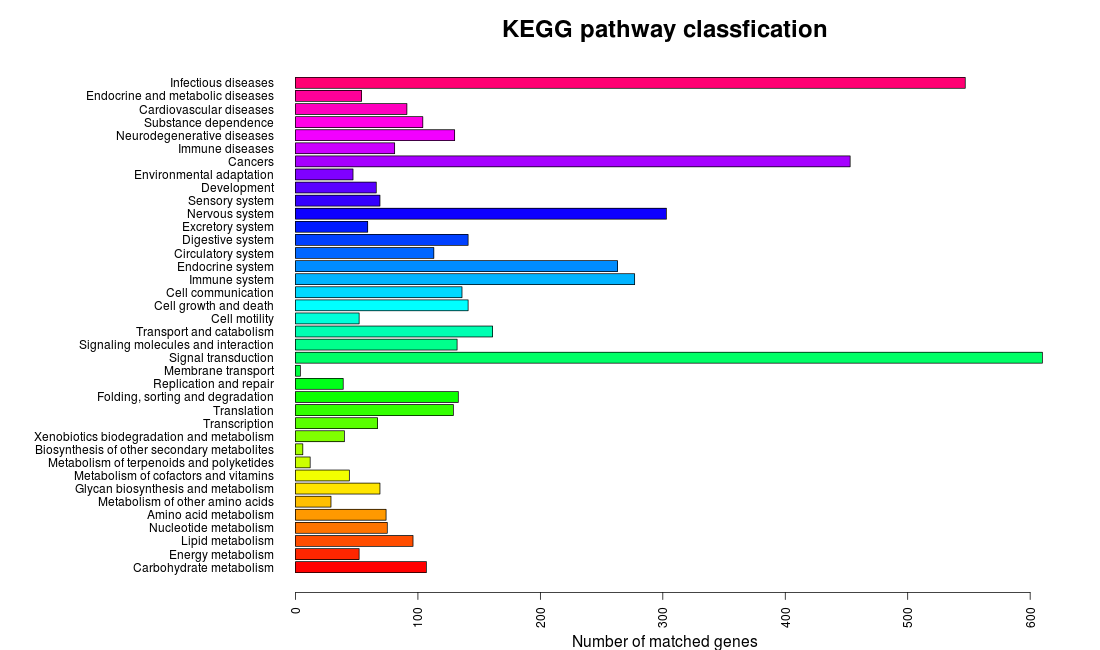

Supplement: Supplementary file 1 [file animals-08-00127-s001.zip › supporting imformation/Figure S5.docx]

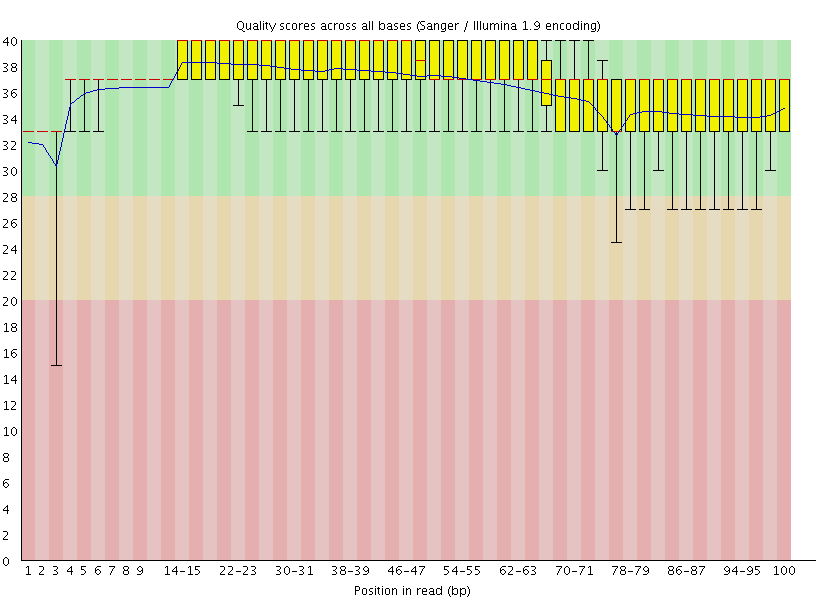

Supplement: Supplementary file 1 [file animals-08-00127-s001.zip › supporting imformation/File.2/Assessment results of FastQC in T1/T-1.R1.per_base_quality.png]

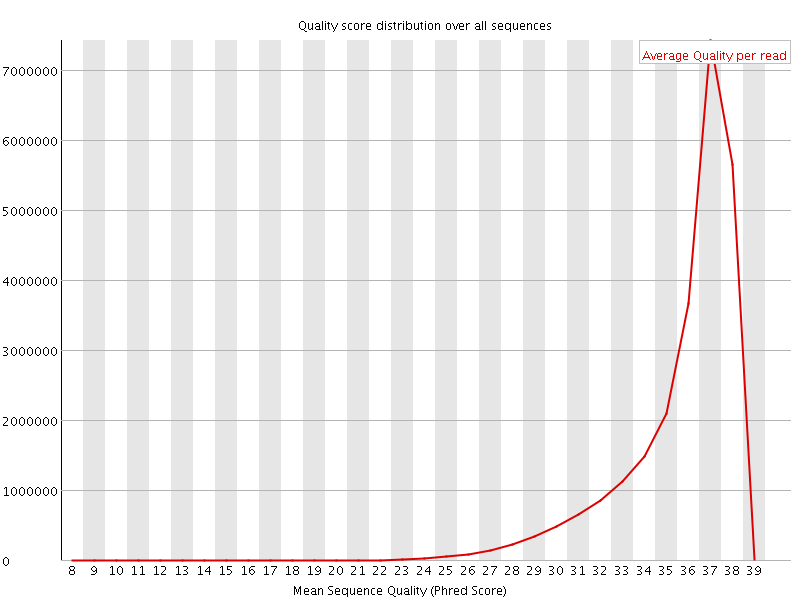

Supplement: Supplementary file 1 [file animals-08-00127-s001.zip › supporting imformation/File.2/Assessment results of FastQC in T1/T-1.R1.per_sequence_quality.png]

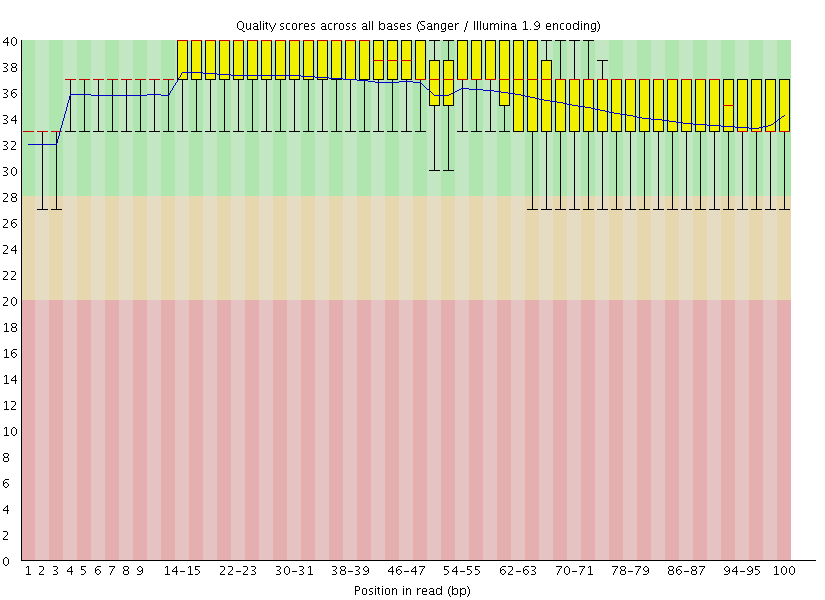

Supplement: Supplementary file 1 [file animals-08-00127-s001.zip › supporting imformation/File.2/Assessment results of FastQC in T1/T-1.R2.per_base_quality.png]

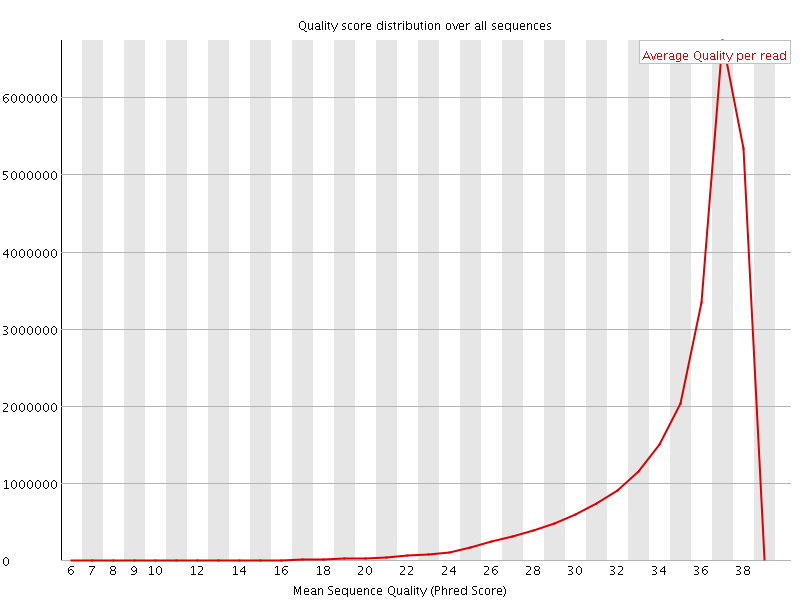

Supplement: Supplementary file 1 [file animals-08-00127-s001.zip › supporting imformation/File.2/Assessment results of FastQC in T1/T-1.R2.per_sequence_quality.png]

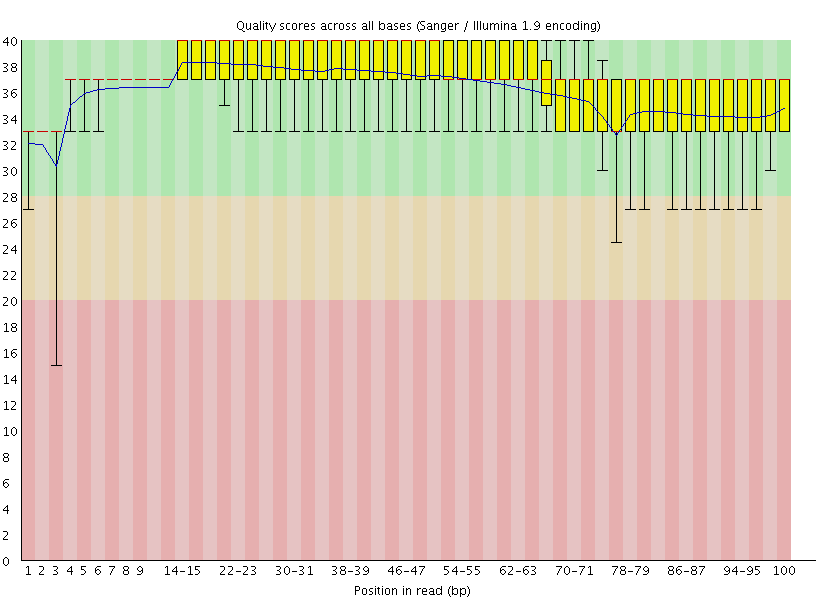

Supplement: Supplementary file 1 [file animals-08-00127-s001.zip › supporting imformation/File.2/Assessment results of FastQC in T2/T-2.R1.per_base_quality.png]

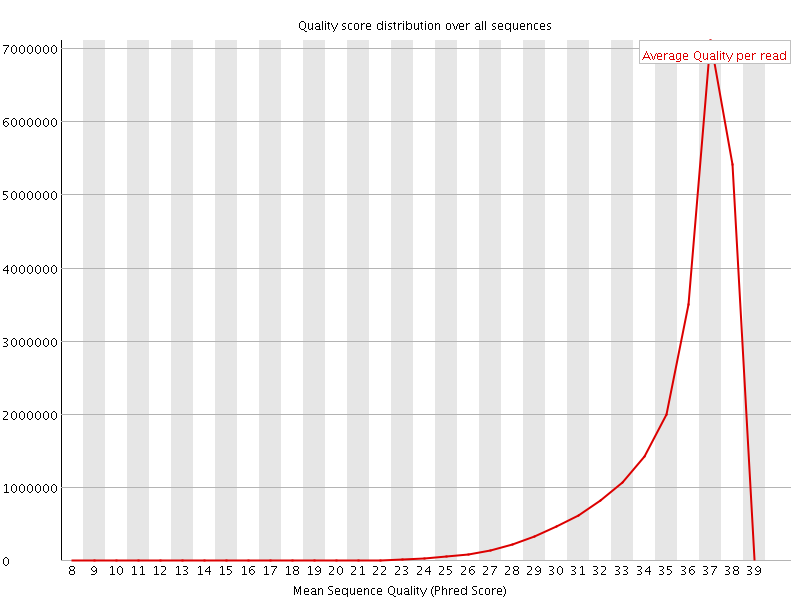

Supplement: Supplementary file 1 [file animals-08-00127-s001.zip › supporting imformation/File.2/Assessment results of FastQC in T2/T-2.R1.per_sequence_quality.png]

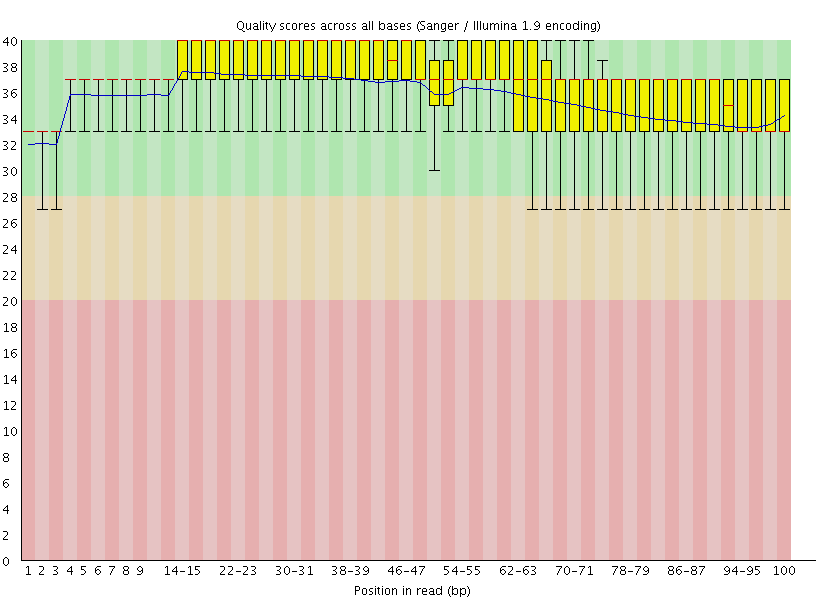

Supplement: Supplementary file 1 [file animals-08-00127-s001.zip › supporting imformation/File.2/Assessment results of FastQC in T2/T-2.R2.per_base_quality.png]

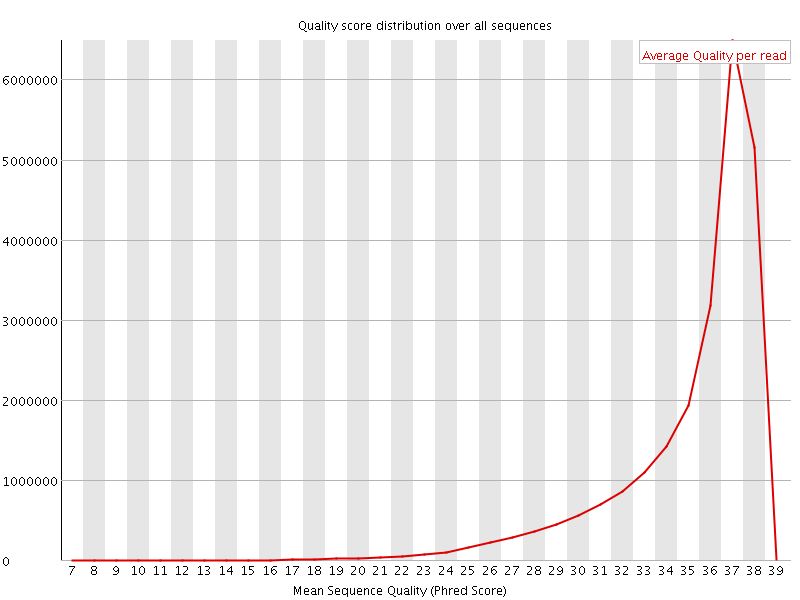

Supplement: Supplementary file 1 [file animals-08-00127-s001.zip › supporting imformation/File.2/Assessment results of FastQC in T2/T-2.R2.per_sequence_quality.png]

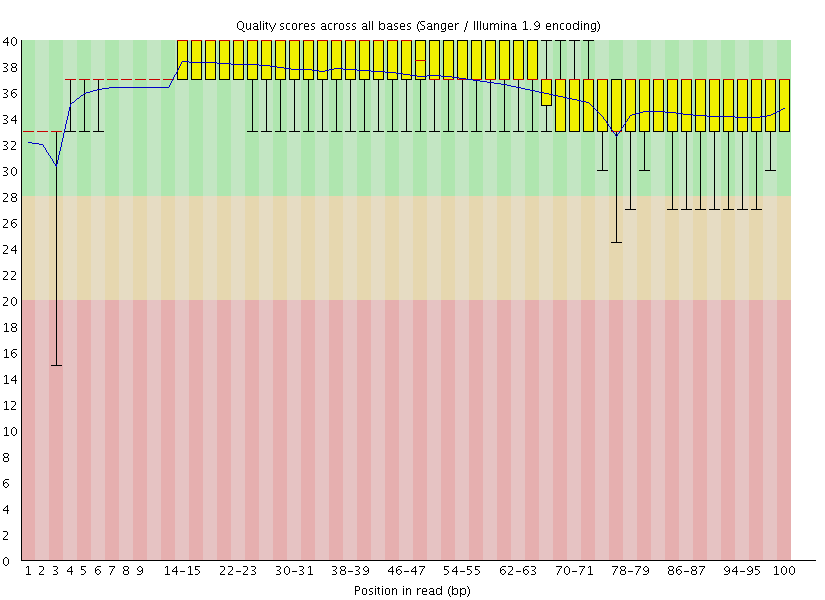

Supplement: Supplementary file 1 [file animals-08-00127-s001.zip › supporting imformation/File.2/Assessment results of FastQC in T3/T-3.R1.per_base_quality.png]

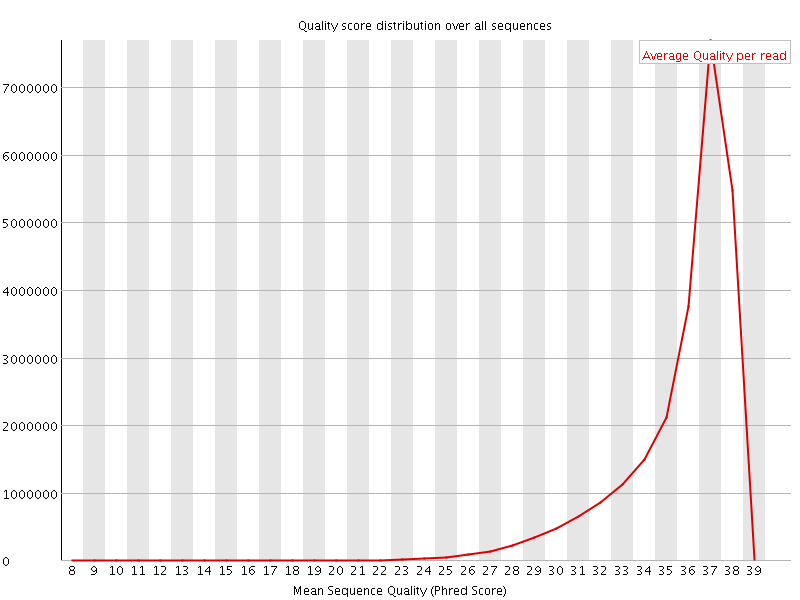

Supplement: Supplementary file 1 [file animals-08-00127-s001.zip › supporting imformation/File.2/Assessment results of FastQC in T3/T-3.R1.per_sequence_quality.png]

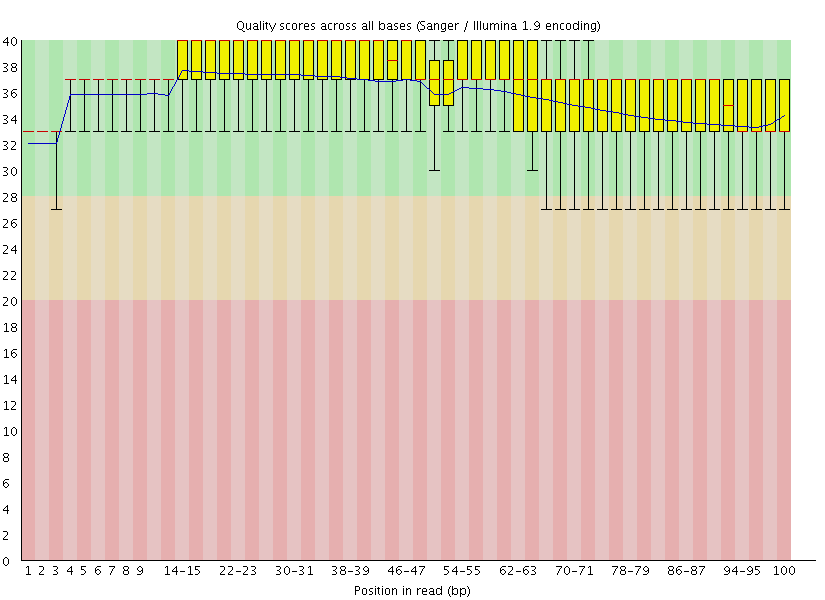

Supplement: Supplementary file 1 [file animals-08-00127-s001.zip › supporting imformation/File.2/Assessment results of FastQC in T3/T-3.R2.per_base_quality.png]

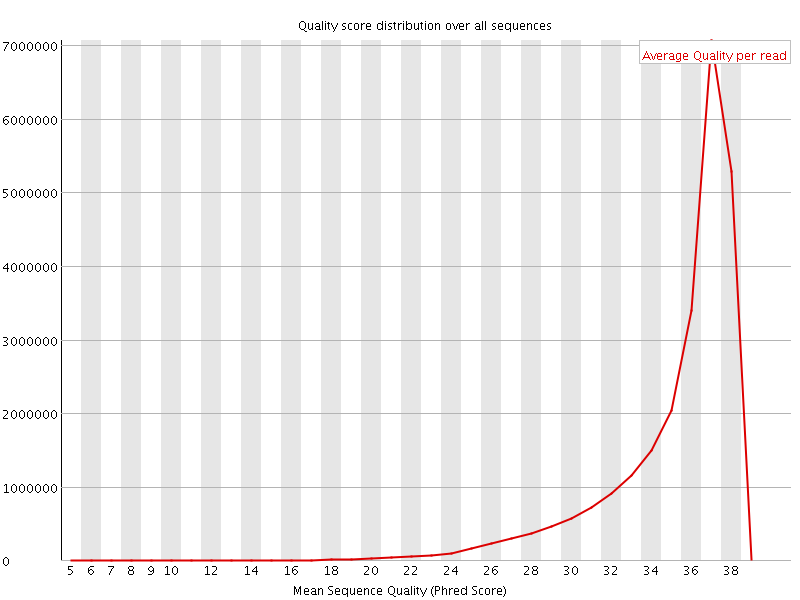

Supplement: Supplementary file 1 [file animals-08-00127-s001.zip › supporting imformation/File.2/Assessment results of FastQC in T3/T-3.R2.per_sequence_quality.png]

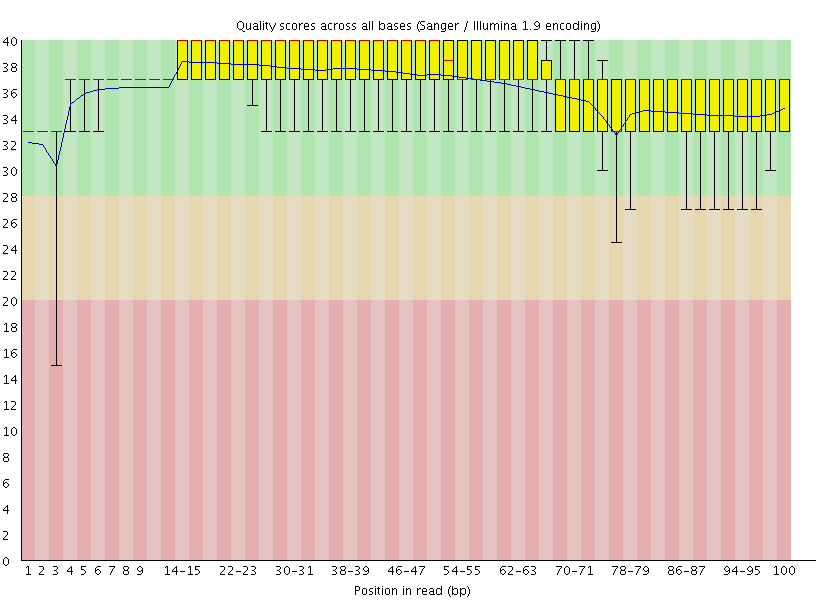

Supplement: Supplementary file 1 [file animals-08-00127-s001.zip › supporting imformation/File.2/Assessment results of FastQC in T4/T-4.R1.per_base_quality.png]

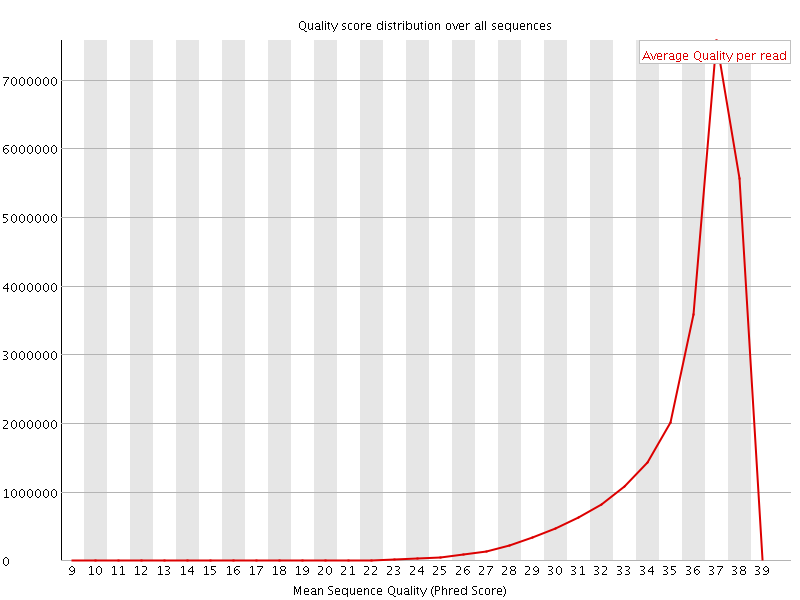

Supplement: Supplementary file 1 [file animals-08-00127-s001.zip › supporting imformation/File.2/Assessment results of FastQC in T4/T-4.R1.per_sequence_quality.png]

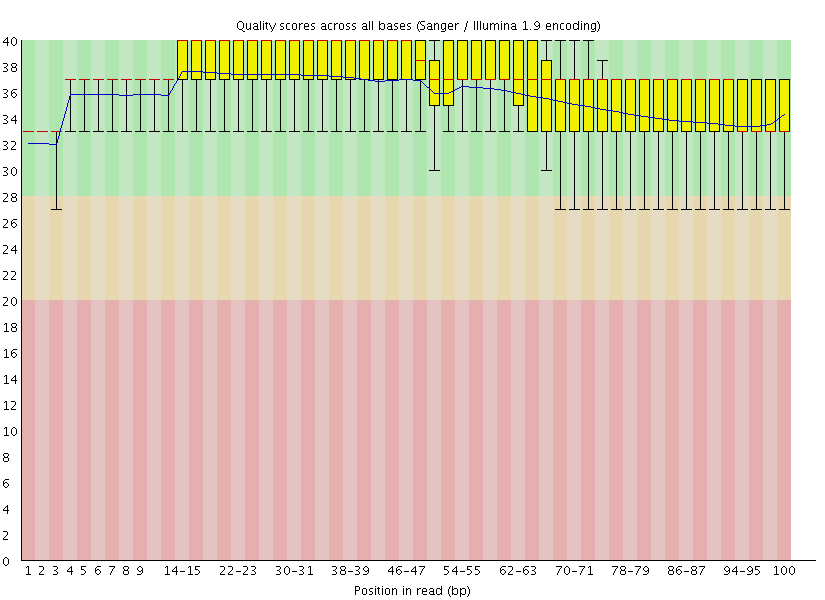

Supplement: Supplementary file 1 [file animals-08-00127-s001.zip › supporting imformation/File.2/Assessment results of FastQC in T4/T-4.R2.per_base_quality.png]

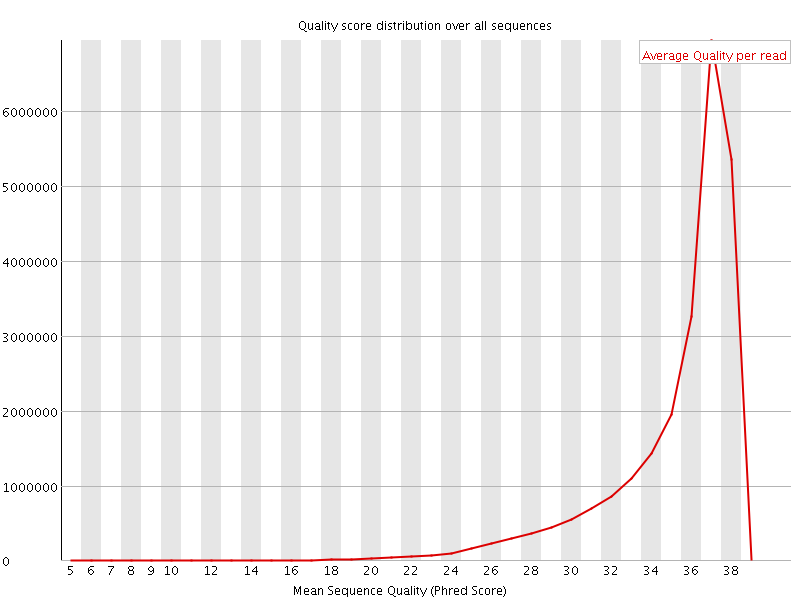

Supplement: Supplementary file 1 [file animals-08-00127-s001.zip › supporting imformation/File.2/Assessment results of FastQC in T4/T-4.R2.per_sequence_quality.png]

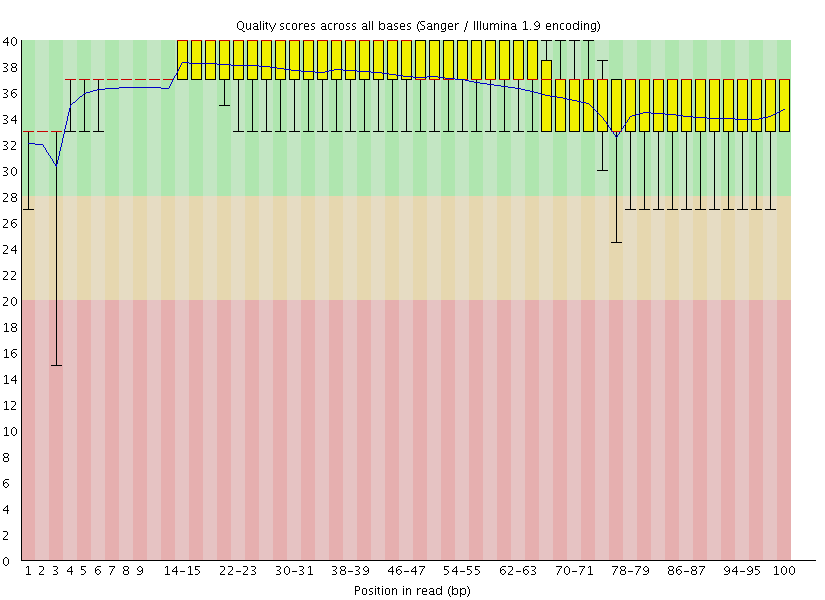

Supplement: Supplementary file 1 [file animals-08-00127-s001.zip › supporting imformation/File.2/Assessment results of FastQC in U1/L-1.R1.per_base_quality.png]

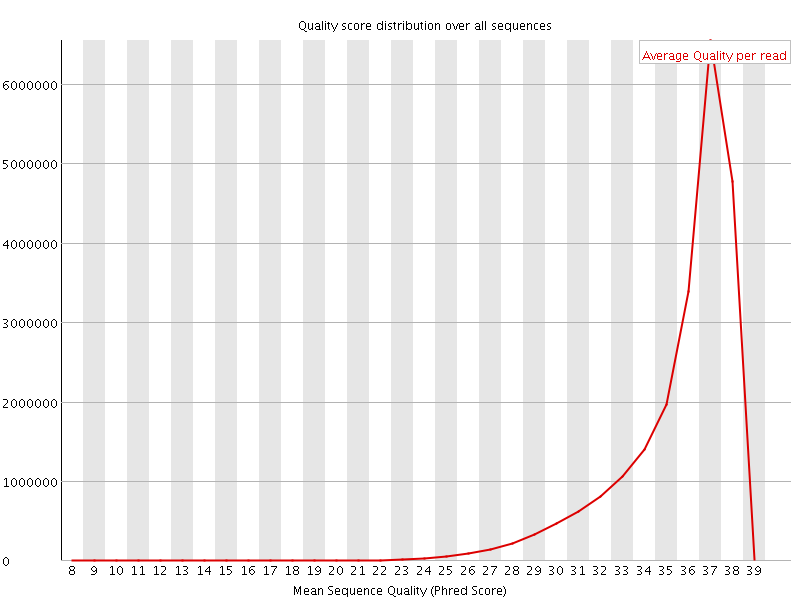

Supplement: Supplementary file 1 [file animals-08-00127-s001.zip › supporting imformation/File.2/Assessment results of FastQC in U1/L-1.R1.per_sequence_quality.png]

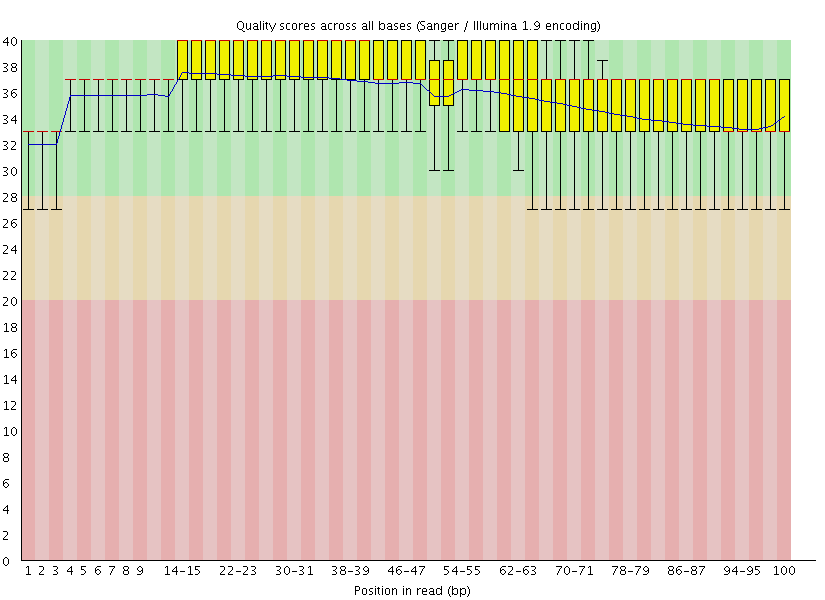

Supplement: Supplementary file 1 [file animals-08-00127-s001.zip › supporting imformation/File.2/Assessment results of FastQC in U1/L-1.R2.per_base_quality.png]

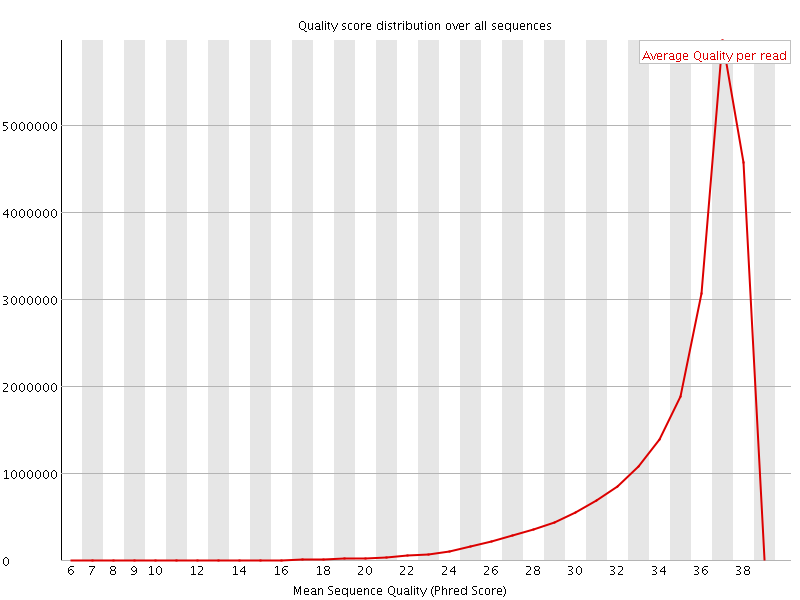

Supplement: Supplementary file 1 [file animals-08-00127-s001.zip › supporting imformation/File.2/Assessment results of FastQC in U1/L-1.R2.per_sequence_quality.png]

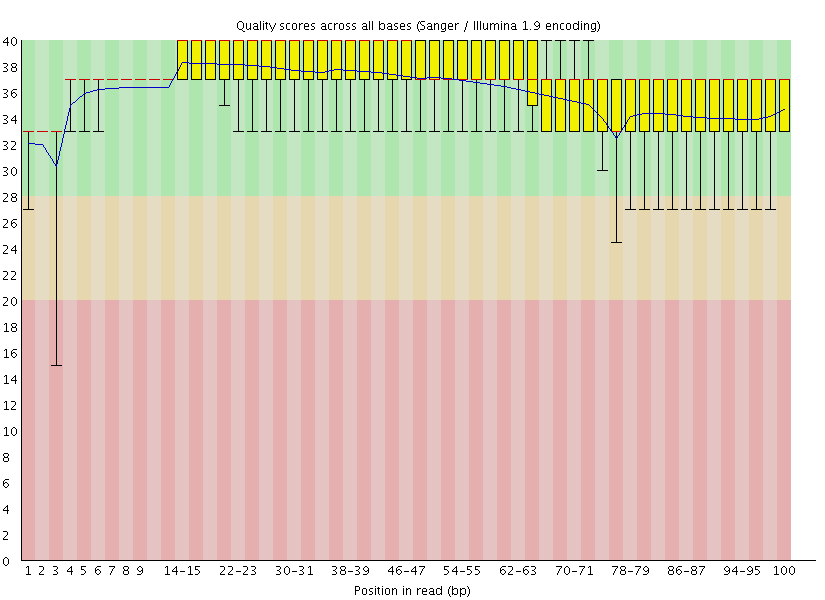

Supplement: Supplementary file 1 [file animals-08-00127-s001.zip › supporting imformation/File.2/Assessment results of FastQC in U2/L-2.R1.per_base_quality.png]

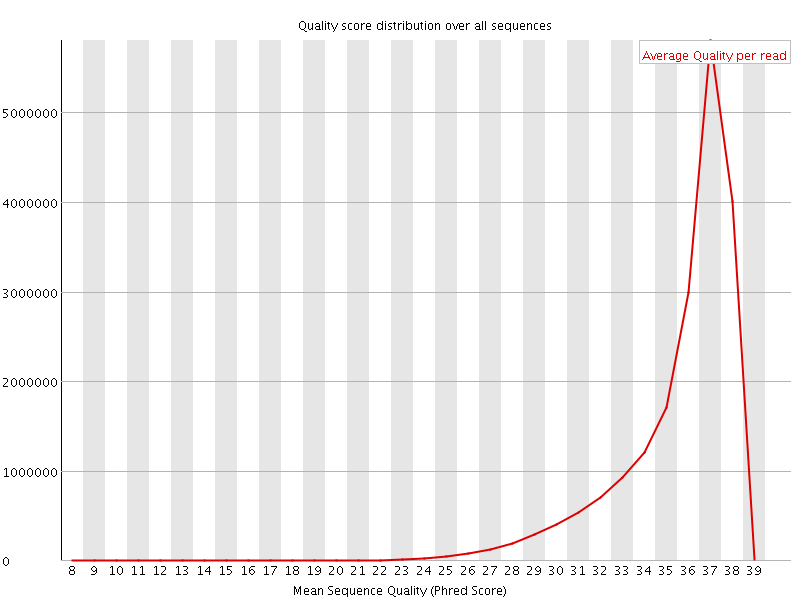

Supplement: Supplementary file 1 [file animals-08-00127-s001.zip › supporting imformation/File.2/Assessment results of FastQC in U2/L-2.R1.per_sequence_quality.png]

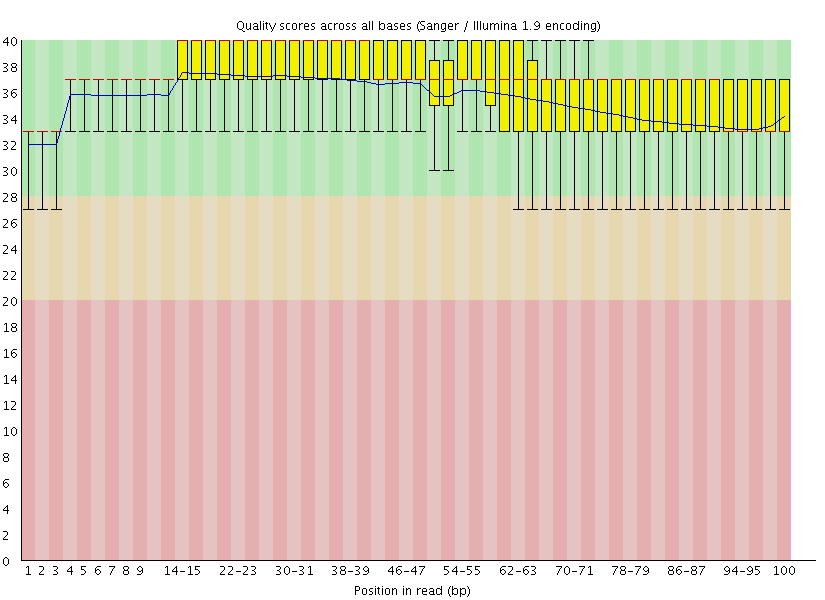

Supplement: Supplementary file 1 [file animals-08-00127-s001.zip › supporting imformation/File.2/Assessment results of FastQC in U2/L-2.R2.per_base_quality.png]

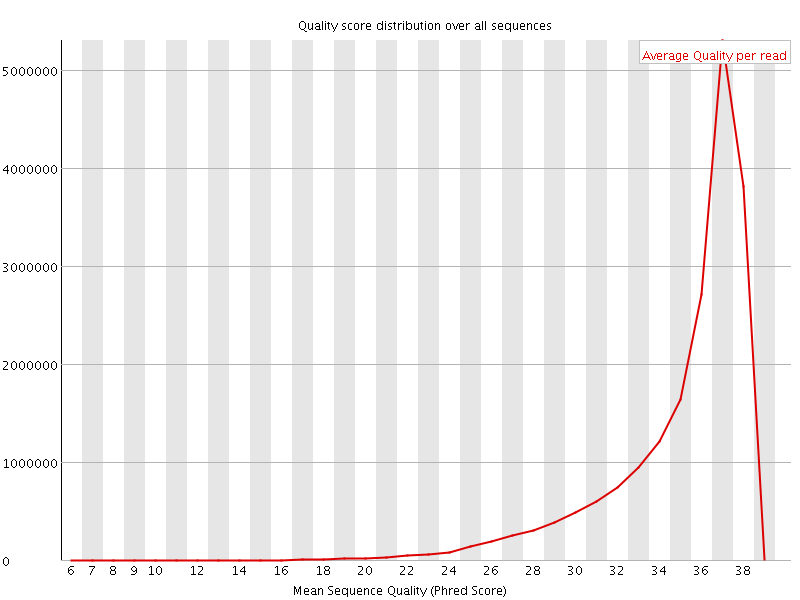

Supplement: Supplementary file 1 [file animals-08-00127-s001.zip › supporting imformation/File.2/Assessment results of FastQC in U2/L-2.R2.per_sequence_quality.png]

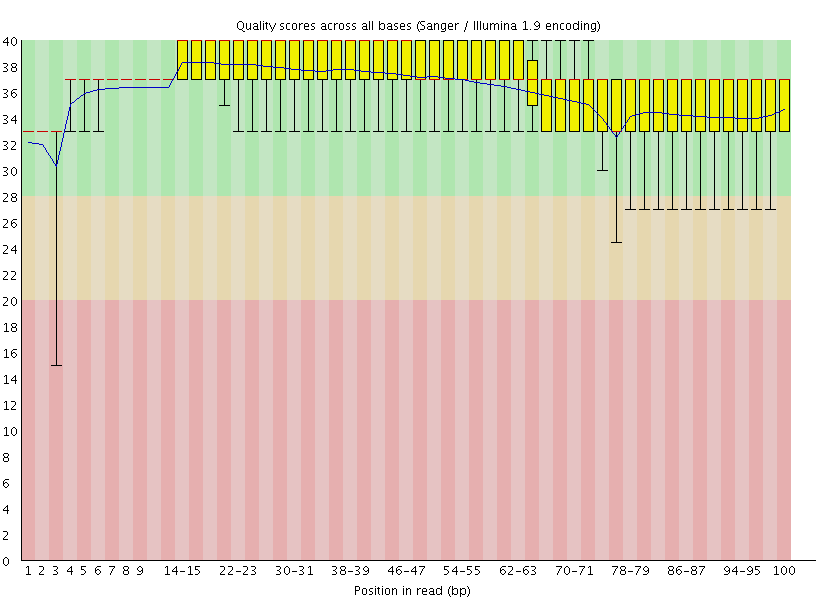

Supplement: Supplementary file 1 [file animals-08-00127-s001.zip › supporting imformation/File.2/Assessment results of FastQC in U3/L-3.R1.per_base_quality.png]

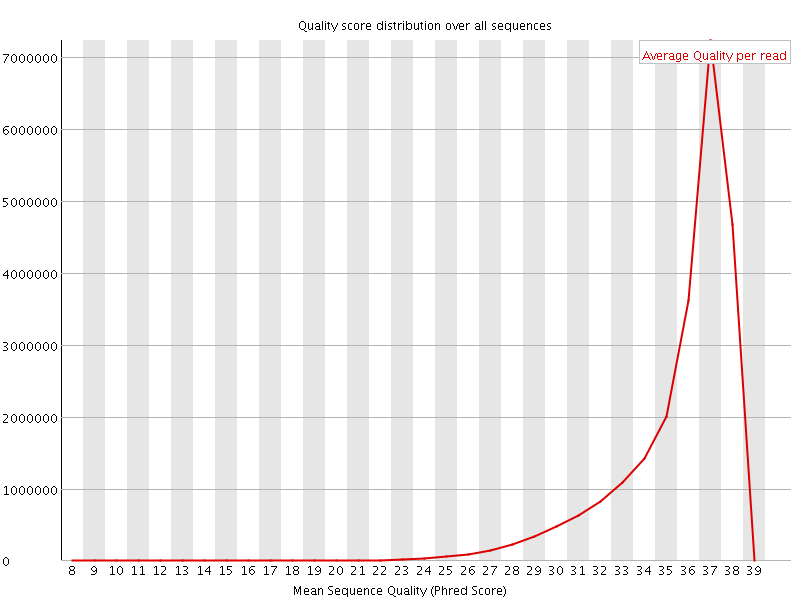

Supplement: Supplementary file 1 [file animals-08-00127-s001.zip › supporting imformation/File.2/Assessment results of FastQC in U3/L-3.R1.per_sequence_quality.png]

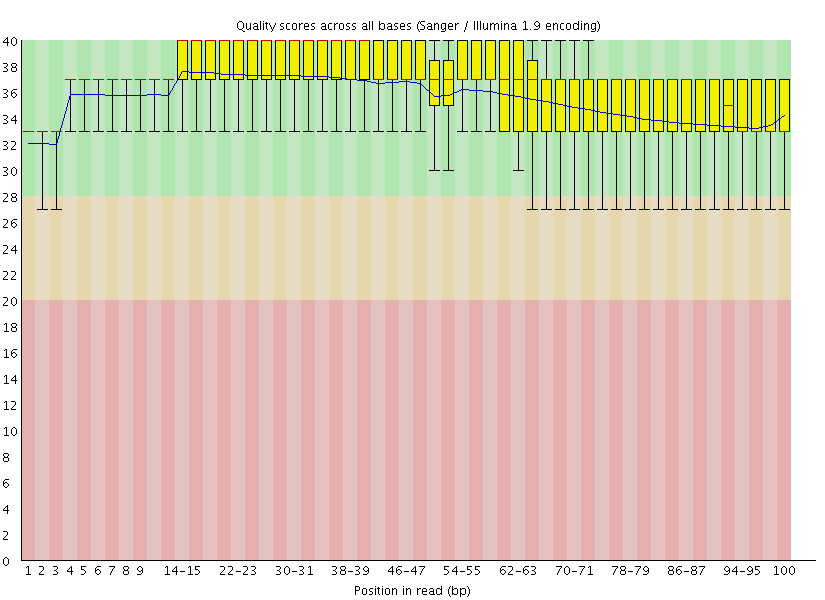

Supplement: Supplementary file 1 [file animals-08-00127-s001.zip › supporting imformation/File.2/Assessment results of FastQC in U3/L-3.R2.per_base_quality.png]

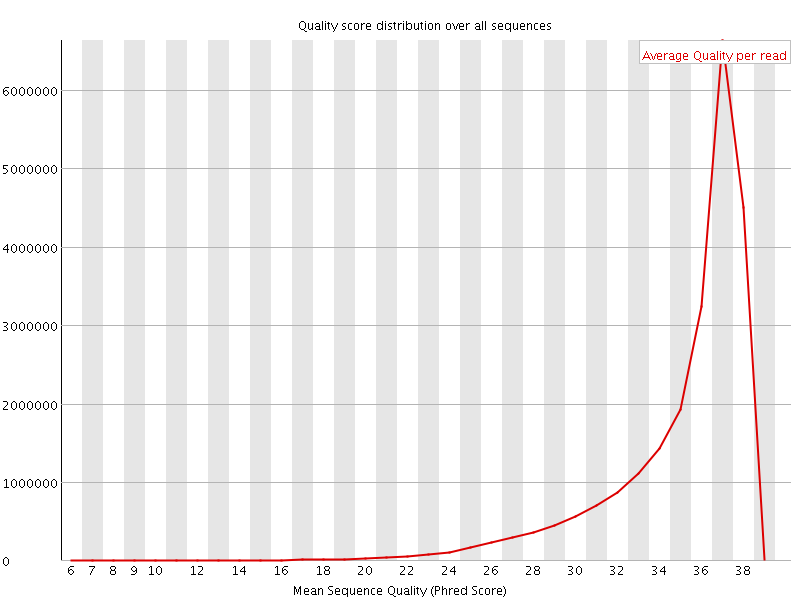

Supplement: Supplementary file 1 [file animals-08-00127-s001.zip › supporting imformation/File.2/Assessment results of FastQC in U3/L-3.R2.per_sequence_quality.png]

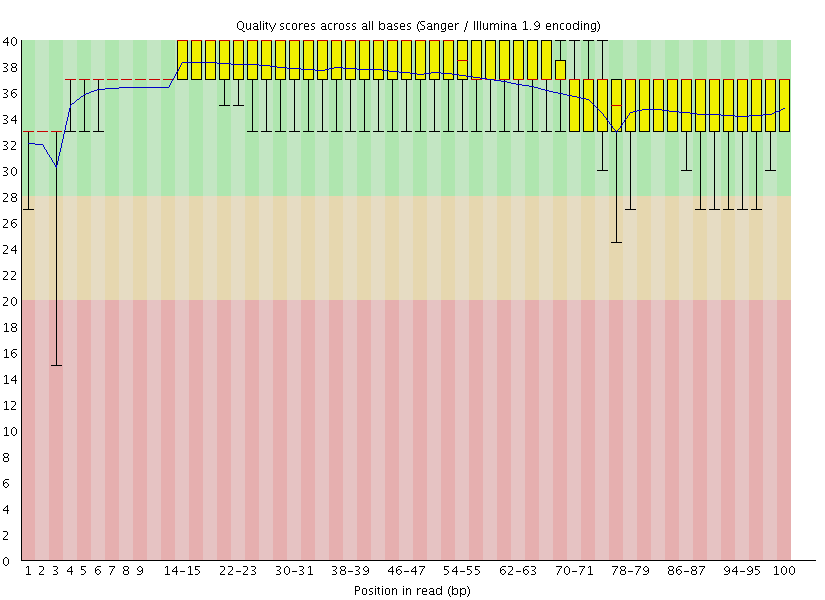

Supplement: Supplementary file 1 [file animals-08-00127-s001.zip › supporting imformation/File.2/Assessment results of FastQC in U4/L-4.R1.per_base_quality.png]

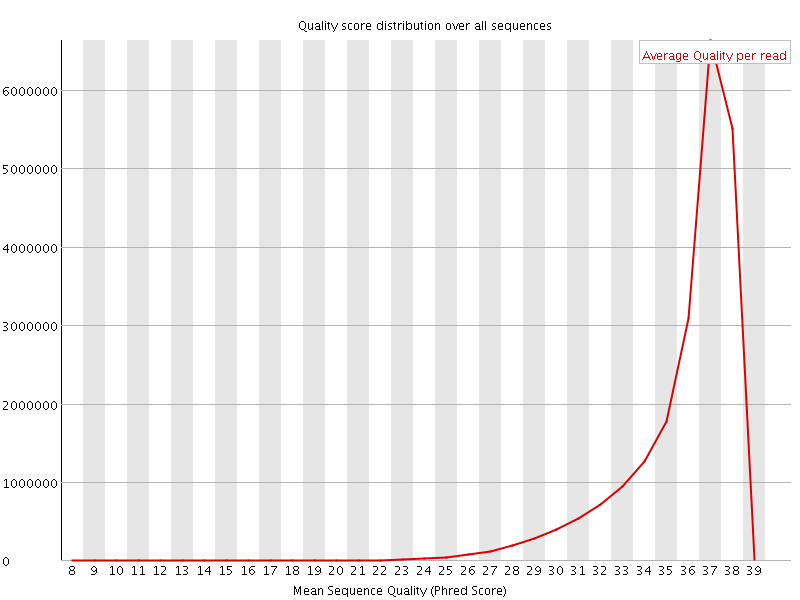

Supplement: Supplementary file 1 [file animals-08-00127-s001.zip › supporting imformation/File.2/Assessment results of FastQC in U4/L-4.R1.per_sequence_quality.png]

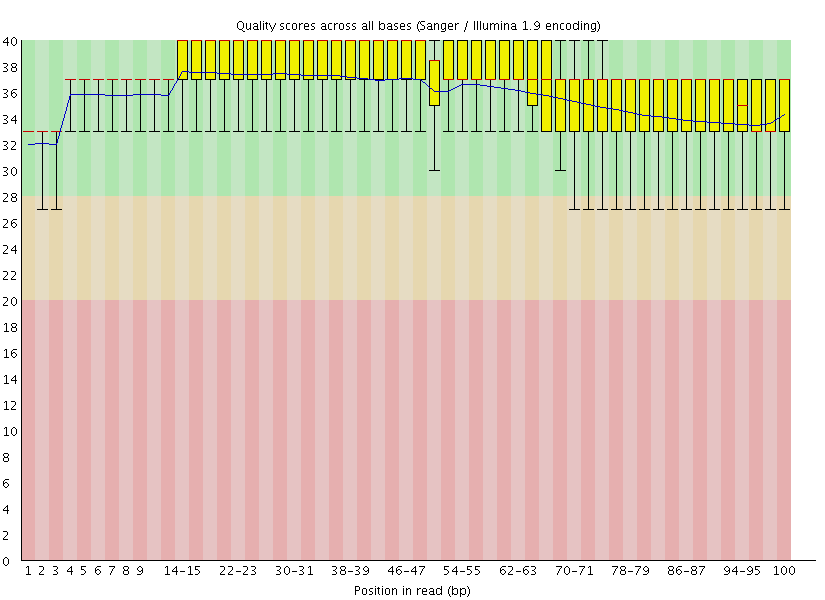

Supplement: Supplementary file 1 [file animals-08-00127-s001.zip › supporting imformation/File.2/Assessment results of FastQC in U4/L-4.R2.per_base_quality.png]

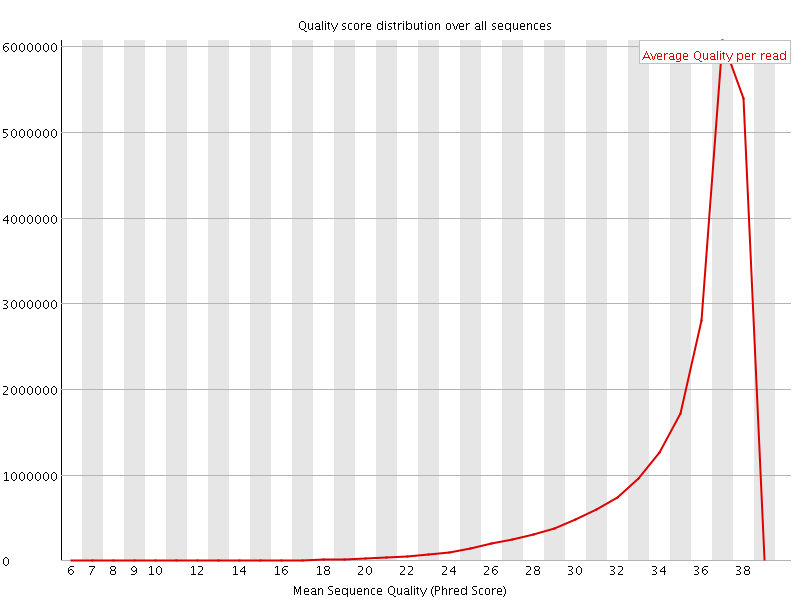

Supplement: Supplementary file 1 [file animals-08-00127-s001.zip › supporting imformation/File.2/Assessment results of FastQC in U4/L-4.R2.per_sequence_quality.png]
